# An infrared spectral biomarker accurately predicts neurodegenerative disease class in the absence of overt symptoms

**DOI:** 10.1038/s41598-021-93686-8

**Published:** 2021-08-02

**Authors:** Lila Lovergne, Dhruba Ghosh, Renaud Schuck, Aris A. Polyzos, Andrew D. Chen, Michael C. Martin, Edward S. Barnard, James B. Brown, Cynthia T. McMurray

**Affiliations:** 1 Division of Molecular Biophysics and Integrated Bioimaging, Lawrence Berkeley National Laboratory, Berkeley, CA 94720 USA; 2 Department of Statistics, University of California, Berkeley, CA 94720 USA; 3 Advanced Light Source, Lawrence Berkeley National Laboratory, Berkeley, CA 94720 USA; 4 Molecular Foundry, Lawrence Berkeley National Laboratory, Berkeley, CA 94720 USA; 5 Division of Environmental Genomics and Systems Biology, Lawrence Berkeley National Laboratory, Berkeley, CA 94720 USA

**Keywords:** Biological techniques, Biomarkers

## Abstract

Although some neurodegenerative diseases can be identified by behavioral characteristics relatively late in disease progression, we currently lack methods to predict who has developed disease before the onset of symptoms, when onset will occur, or the outcome of therapeutics. New biomarkers are needed. Here we describe spectral phenotyping, a new kind of biomarker that makes disease predictions based on chemical rather than biological endpoints in cells. Spectral phenotyping uses Fourier Transform Infrared (FTIR) spectromicroscopy to produce an absorbance signature as a rapid physiological indicator of disease state. FTIR spectromicroscopy has over the past been used in differential diagnoses of manifest disease. Here, we report that the unique FTIR chemical signature accurately predicts disease class in mouse with high probability in the absence of brain pathology. In human cells, the FTIR biomarker accurately predicts neurodegenerative disease class using fibroblasts as surrogate cells.

## Introduction

Although disease-causing mutations are well known, the vast amount of available data have not necessarily led to robust disease detection, or to a good understanding of disease etiology, particularly for neurodegeneration. The identification of reliable disease biomarkers has been difficult and hindered by the fact that the brain is not an accessible tissue. Thus, classification relies on clinical diagnosis, which is not always certain^1,2^. Alzheimer’s disease (AD) and Huntington’s disease (HD) provide good examples. HD and AD are typically late onset diseases^3^, which arise from neuronal loss in the striatum and hippocampus, respectively. However, the former is a dominant single-gene defect, while the underlying genetic causes of the latter are unknown for 95% of patients^3–5^. There is no way to predict in advance who will develop AD or its onset. Moreover, the characteristic cognitive decline is not unique to AD and can occur during normal aging^1^. Although a battery of neuropsychological tests is often used in making a clinical diagnosis of AD, a definitive diagnosis still relies on pathological evaluation of plaques and tangles at autopsy^2^.

HD is characterized by motor decline^6,7^, striatal death^8,9^ with well-defined genetics. The underlying mutation in HD is expansion of a CAG triplet repeat tract in exon 1 of the expressed disease allele^10–12^. Using traditional genetic screens, the onset of HD is predictable by the length of the CAG repeat tract. The longer the tract, the more severe is the phenotype. However, there are unknown modifier genes^13–15^ whose effects vary with the patient. While the onset of HD patients with a CAG tract of 50 is on the average around 50 years of age^16^, the onset of any particular patient with a repeat tract length of 50 can vary as much as fourfold, ranging from 20 to 80 years^16^. Thus, quality of life can differ significantly among HD patients of the same repeat tract length, but disease outlook is not always certain. The pathology in a brain section is obvious for an HD or an AD patient after death, and biomarkers are not needed to make a postmortem diagnosis. However, an early biomarker to predict disease during life would be a significant advance.

**Figure 1:**
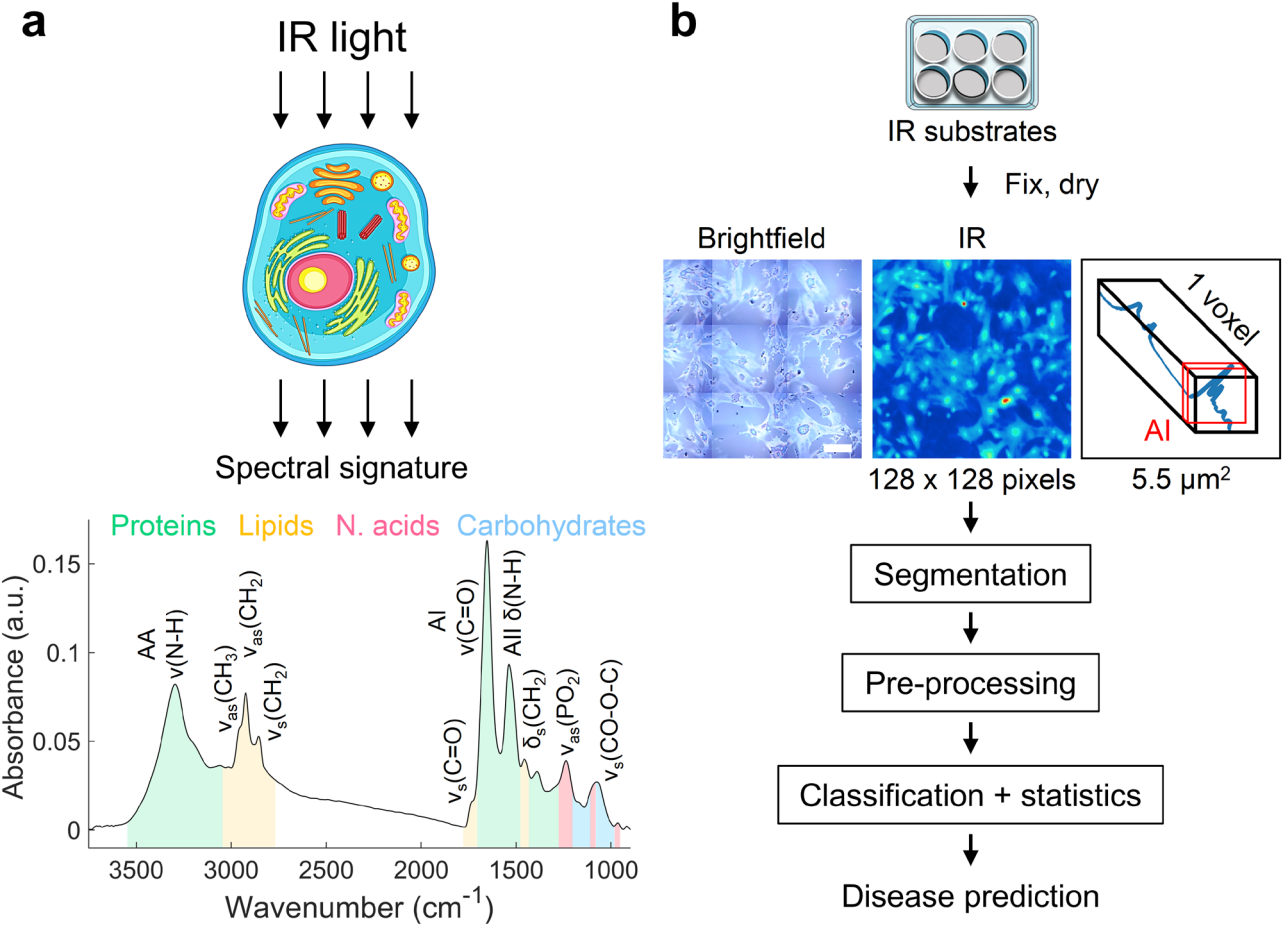
Concept of cell phenotyping by infrared spectroscopy. (**a**) Schematic of a representative infrared spectrum of astrocytes and the attribution of the prominent chemical features between 4000 and 900 cm^−1^. AA/I/II: amide A/I/II, ν: stretching, δ: bending, as: asymmetric, s: symmetric vibrations. (**b**) Brief outline of the analysis pipeline for spectral phenotyping, as discussed in the text. After 7–10 days, cells are plated and cultured overnight onto IR compatible calcium fluoride (CaF_2_) substrates, fixed and dried before the spectral analysis. A representative brightfield and corresponding IR image of astrocytes are displayed. IR images are reconstructed on the amide I band (AI) for optimal background/cell contrast. Each tile comprises 128 by 128 pixels (5.5 µm^2^), each of which contains a FTIR spectrum (in blue), thus constituting hyperspectral images. The raw spectral images are carried through three processing steps to generate a cell signature. (Segmentation) The cells are segmented to extract from IR images the nucleus, cytoplasm, and whole cell raw spectra. (Pre-processing) Raw spectra are pre-processed to generate normalized second derivative spectra. (Classification and statistics) Statistical analysis is used to evaluate the disease classification using Principal Component Analysis (PCA) and Uniform Manifold Approximation and Projection (UMAP) analysis. Scale bar = 100 µm.

Towards this effort, we have developed a general-use Fourier Transform Infrared (FTIR) technology which predicts disease class with high probability. Over the years, FTIR as well as Raman microspectroscopies have emerged as useful tools for characterization of biological samples based on their unique chemistry and spectral properties (Fig. 1a)^17–25^. Indeed, infrared irradiation produces an absorbance spectrum that integrates the vibrational state of tens of thousands of endogenous chemical features (Fig. 1a)^26–28^. The resulting absorbance spectrum does not correspond to a single molecule. Rather, it is an integrated physiological “read-out” of all molecular bonds originating from the function groups in proteins, lipids, carbohydrates, and nucleic acids^26^. While all cells have the same collection of functional groups, band intensity and position will vary depending on the group’s abundance, hydrogen bonding, bond angle, and molecular context^19,20,28,29^. Thus, the composition of the FTIR signature fingerprints cells^17,18^ (Fig. 1a). The FTIR absorbance profile is a powerful discriminator since it is based on whole-cell chemistry rather than on specific biological endpoints or single point markers^30^. Thus, the change in an FTIR absorbance spectrum reflects real physiological changes such as those that accompany a disease.

Based on its chemical richness, FTIR has been used successfully for differential diagnosis of cancer subtypes in patients with manifest disease^31,32^, attesting to its powerful discrimination capability. However, our approach goes one step further in asking whether (1) a spectral phenotyping approach is capable of robust classification of neurodegenerative disease before the manifestation of overt symptoms in a mouse astrocyte model, and (2) whether disease prediction is possible using non-neuronal human cells as surrogates. These are important capabilities since the human brain is not accessible during life and biological symptoms may occur too late in patients for effective therapeutics.

Here, we report the development of spectral phenotyping, a reliable algorithm to predict disease and non-disease classes. Both a standardized analytical approach^22,33–35^ and best practice metrics^36–41^ are critical parameters and are described for our analysis. The strategy followed a two-step plan: (1) to develop a robust algorithm using a stable mouse system with little biological variation, and (2) to test the prediction algorithm with more variable human HD or AD fibroblasts, which were used as brain cell surrogates. For the mouse experiments, the FTIR biomarker was benchmarked using a well characterized *HdhQ(150/150)* inbred model of HD and compared to its genetically matched control strain, C57Black6 (*C57Bl6J*), which do not express the mutant gene. The *HdhQ(150/150)* line harbors an expanded CAG repeat tract of 150 knocked into the endogenous mouse Huntington gene locus^42^. The *HdhQ(150/150) *line is a good model for “late onset” disease, since these animals express the mutant huntingtin (mhtt) disease protein at physiological levels from birth but do not display symptoms until late in life^42,43^. Thus, HD animals from 2 days to 2 years were tested to assess the likelihood that an early disease prediction by FTIR spectroscopy was possible in the absence of a disease phenotype. Spectral phenotyping was not only successful in disease classification in the absence of overt pathology in the mouse model, but also predicted neurodegenerative disease class in HD and AD patients using fibroblasts as surrogates for brain cells.

## Results

FTIR signatures were acquired by mid-IR range light (wavelengths from 2.5 to 25 µm)^26–28^ and measuring the absorbance profile of vibrational frequencies (wavenumbers in cm^−1^) between 4000 and 900 cm^−1^ (Fig. 1a). The astrocytes were cultured on IR transparent calcium fluoride substrates (Fig. 1b), and a user-defined number of adjacent field of views (FOV) were exposed to IR light. Their IR absorption spectra were collected at multiple wavelengths using a focal plane array (FPA) light detector, which is placed in the image plane of the microscope (Fig. 1b). Within the 128 by 128 pixel FOV, each pixel (set to 5.5 µm^2^) of the hyperspectral image contains a complete FTIR absorbance spectrum (Fig. 1b), which is processed to obtain the chemical signature for the cells. The steps of sample preparation, FTIR acquisition, image segmentation, analysis, and statistical pipeline (Fig. 1b) will be briefly discussed in the text, and the details are provided in the methods section.

### Early postnatal astrocytes from WT and HD mice are indistinguishable by obvious criteria

**Figure 2:**
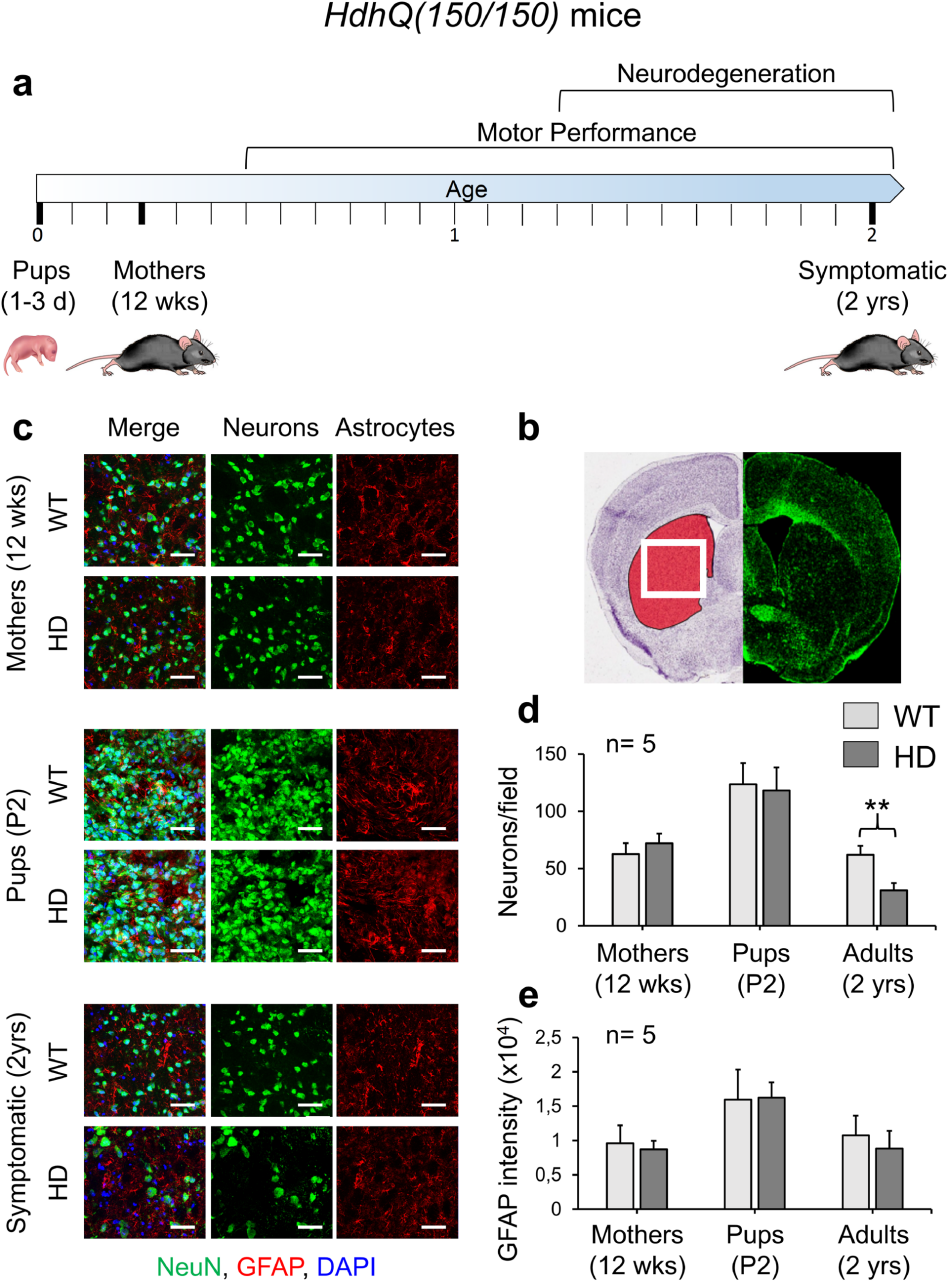
HD mothers and their pups display no overt pathology relative to WT animals. (**a**) Schematic summary of behavior in *HdhQ(150/150)* animals with age^42,43^. The P2 pups, their mothers (12 weeks), and symptomatic 2-year animals are displayed on the timeline. (**b**) Cartoon adapted from the Allen Reference Atlas depicting an adult striatum in red and the white box indicating the regions probed in the brain slices in (c); (**c**) Mouse striatal brain sections were analyzed for neurons (NeuN antibody) alone, astrocytes (GFAP antibody) alone or as a merged image (Merge) of the two. The striatal regions were compared between WT and HD animals at various ages. There are no differences in neuronal counts in the striatum of HD animals compared to WT, except at very late ages (2 years of age). There is no significant difference in astrocyte levels (GFAP intensity per field) between HD and WT at any age. (**d**) Quantification of neuronal counts and astrocyte counts from (**c**). **p value < 0.005 (Student’s *t*-test, 2 tailed, equal variance homoscedastic). Scale bar is 50 µm.

We implemented spectral phenotyping for robust disease predictions in astrocytes isolated from *C57Bl6J* or *HdhQ(150/150)* animals^42,43^, which are referred to throughout as wild-type (WT) and HD, respectively. HD pathology was evaluated in brain sections from newborn pups at postnatal day 1–3 (referred to as P2) (Fig. 2a), in 12-week mothers, and in 2 year affected animals to establish the earliest non-symptomatic age window for FTIR analysis. The brains of the P2 pups displayed no obvious pathology (Fig. 2c–e). Indeed, pups of both genotypes had a similar number of neurons in the striatum (Fig. 2b), the region most prone to neural death in HD. As quantified by the NeuN antibody marker for neurons (Fig. 2c,d) and the astroglial marker, Glial Fibrillary Acidic Protein (GFAP) (Fig. 2c,e), there were no measurable changes in cell morphology or number in the brains of P2 animals. Similarly, the 12-week mothers also showed no brain pathology (Fig. 2c–e) and were asymptomatic by standard measures of motor function compared to WT animals of similar ages (Fig. 2a; Supplementary Fig. 1a). This was in contrast to 2-year HD animals, which had lost roughly 50% of their neurons relative to WT animals of comparable age (Fig. 2c,d) and had developed substantial motor dysfunction^42,43^ (Fig. 2a; Supplementary Fig. 1a). Collectively, WT and HD P2 pups differed in genotype but were not distinguishable by overt phenotypes.

**Figure 3:**
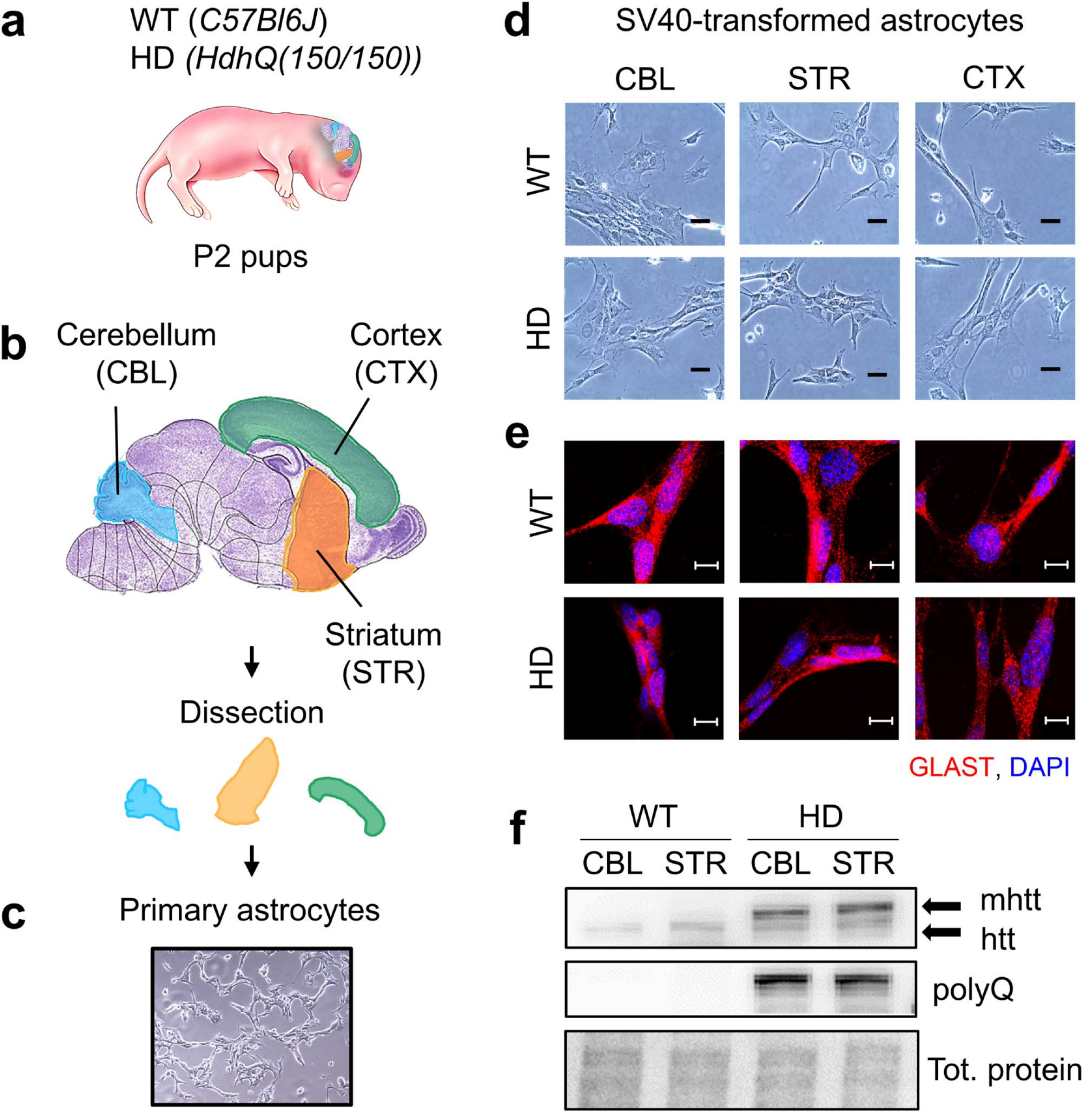
Astrocyte cultures from WT and HD animals are visually indistinguishable. (**a**) Astrocyte cell lines from CBL, STR, CTX were dissociated and isolated from the brains of postnatal (P2) mice, from either WT or HD mice. (**b**) Cartoon adapted from the Allen Reference Atlas for the developing mouse brain at P4 and the dissected regions used in the analysis. The regions are schematically illustrated is the Nissl-stained brain image (purple) from P4 animals. (**c**) A representative brightfield image of primary astrocytes from the cortex of WT mice. (**d**) Purified SV40T astrocytes in all 3 brain regions from WT and HD mice. Scale bars = 20 µm. (**e**) Transformed cultures were stained for Glutamate Aspartate Transporter 1 (GLAST1) antibody marker to confirm their identity as astrocytes, as well as stained with DAPI to define the nucleus. Scale bars = 20 µm. Cell lines of either genotype have similar morphology. (**f**) Western blot analysis showing that mouse astrocytes from WT and HD mice express normal htt and the mutant (mhtt), respectively, in the STR and CBL. HD astrocytes alone express mhtt, which includes an expanded polyQ stretch. The loading control is total protein visualized with No-Stain Protein Labelling Reagent. The uncropped images are shown in Supplemental Fig. 1c.

We purified astrocytes from P2 pups and evaluated whether cells from WT and HD animals could be distinguished by visual cues in culture. A cartoon adapted from the Allen Mouse Brain (P4) Atlas highlights the three brain regions dissected for preparation of astrocytes; the striatum (STR) is the most susceptible region, the cortex (CTX), and the cerebellum (CBL), which is most resistant to neurodegeneration (Fig. 3a,b). After dissection, the isolated astrocytes from each region (Fig. 3c) were immortalized with simian virus large T antigen (SV40T), as described in methods. The transformed cells provided clonally derived, continuous astrocyte lines to minimize batch effects. The WT and HD cells in culture were indistinguishable. They had similar morphology as illustrated by the bright field (Fig. 3d) or immunofluorescence images (Fig. 3e) and had an equivalent number and activity of mitochondria, which were reflected in the intensity of Mitotracker Green signal (Supplementary Fig. 1b). Indeed, there were no region-specific differences that were obvious by eye in any of the lines and all stained positively for Glutamate Aspartate Transporter 1 (GLAST1) (Fig. 3e), establishing their identity as astrocytes. Although the astrocyte cell lines from WT and HD animals retained expression of the huntingtin (htt) or mhtt protein, respectively (Fig. 3f, shown are CBL and STR: Supplementary Fig. 1c), there were no physical cues to classify these cells as normal or disease. Thus, we tested whether their chemistry, as judged by the FTIR spectral signature, could accurately predict the disease class of these astrocytes isolated at presymptomatic stages.

### Cell segmentation increased the accuracy of predictions

**Figure 4:**
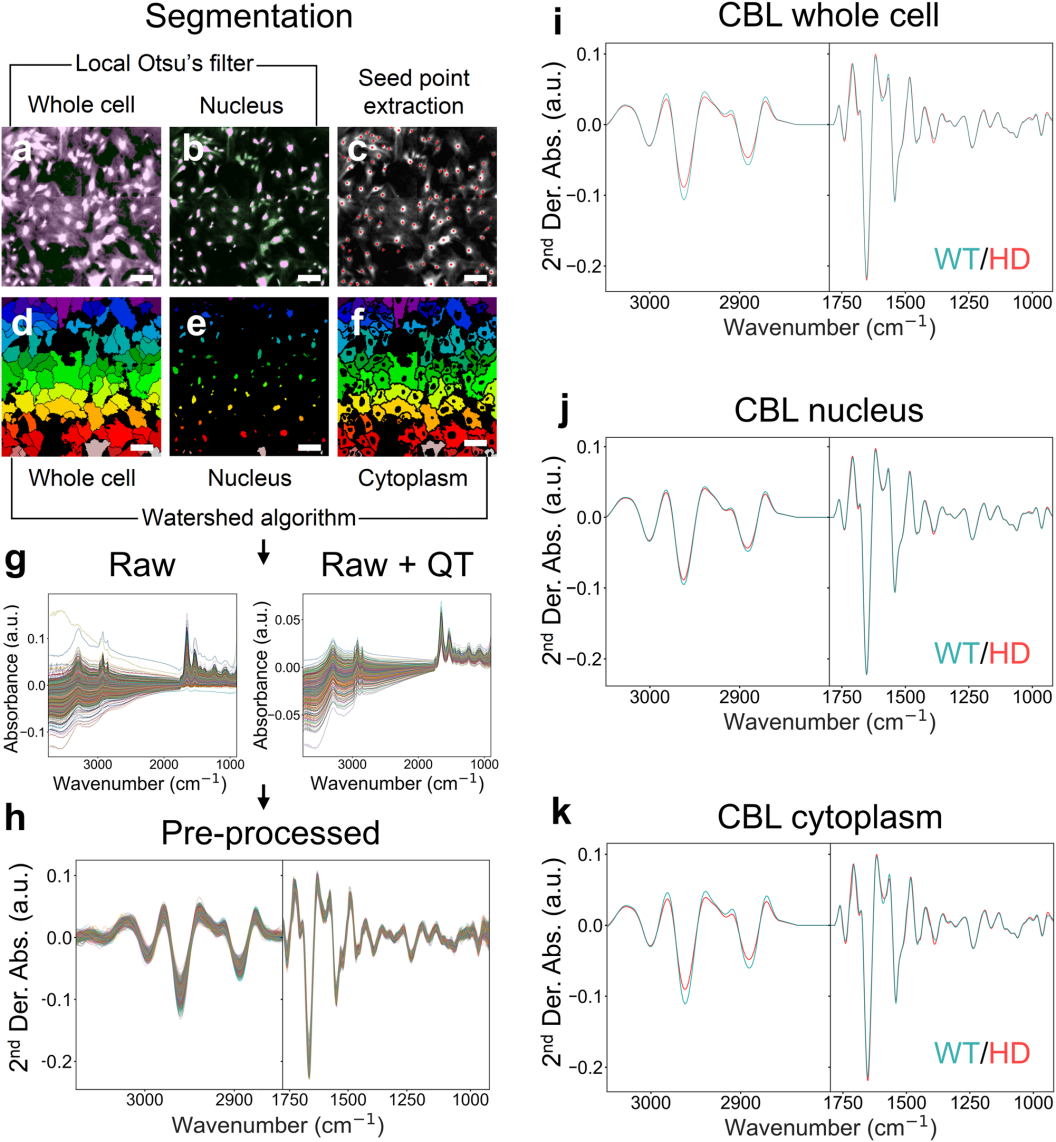
Segmentation reveals differences in the lipid features in the WT and HD astrocytes FTIR signatures. (**a**) Local Ostu’s filter is applied to determine the background from the entire cell or (**b**) nucleus (shown in magenta). (**c**) Seed points are used to localize cells from their estimated center (red dots). (**d**) Seed watershed segmentation applied to whole cells and (**e**) nuclei. (**f**) Watershed segmentation applied to the cytoplasm of the cells (entire cell pixels minus nucleus pixels). Scale bars = 100 µm. (**g**) An example of raw extracted whole astrocyte mean spectra before (left) and after (right) quality testing (QT) and (**h**) pre-processing. (**i**) Whole cell, (**j**) nucleus, and (**k**) cytoplasm average spectra of WT and HD SV40T CBL astrocytes. For visual purpose 2^nd^ derivative normalized spectra are displayed between 3050 and 2800 cm^−1^ (lipid-rich region) and 1800–900 cm^−1^ (“fingerprint” region).

**Figure 5:**
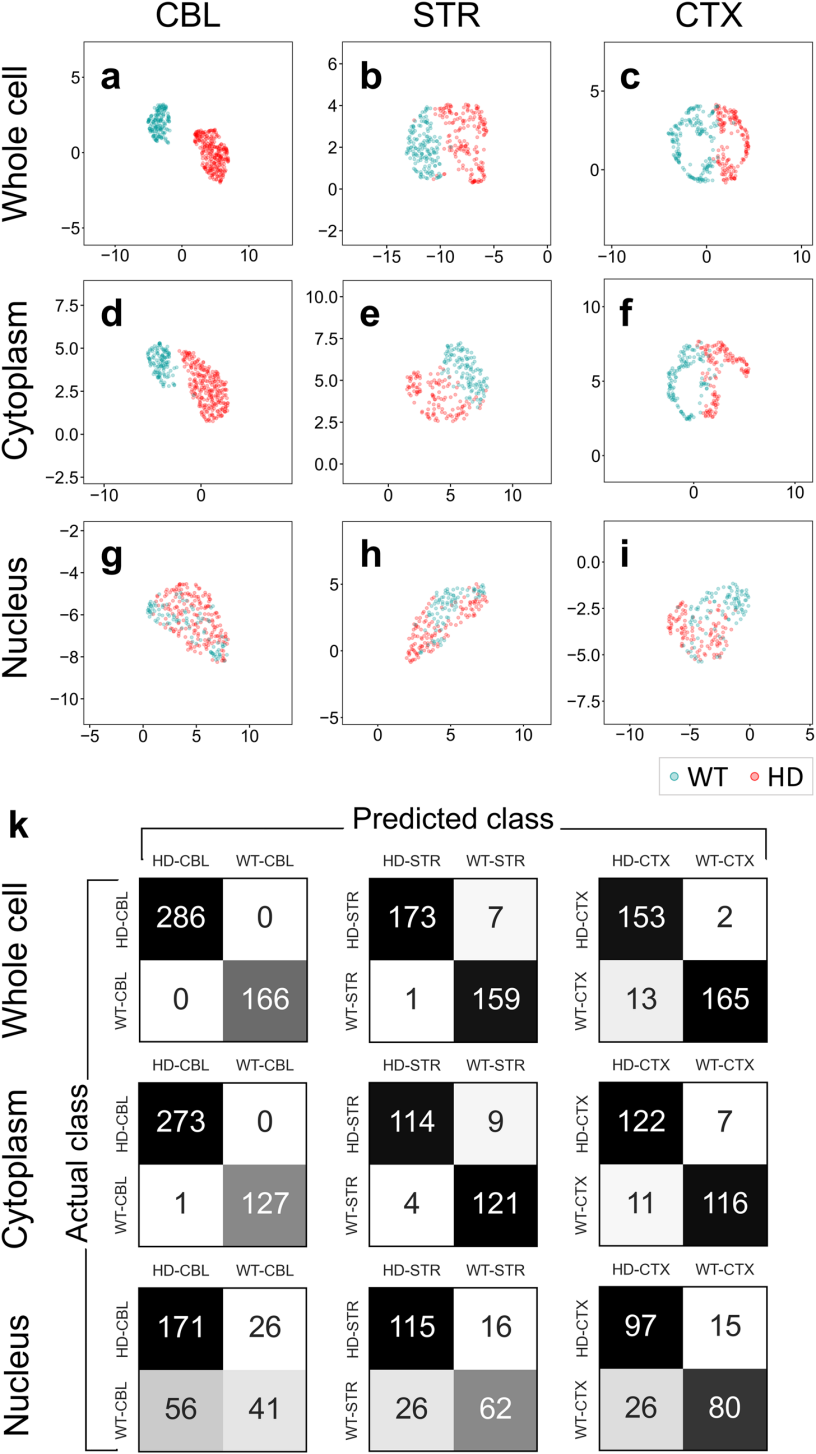
Spectral phenotyping accurately predicts disease class in HD astrocytes. UMAP clustering and classification derived from segmented whole cell (**a**–**c**), cytoplasm (**d**–**f**) or nucleus (**g**–**i**) for three regions of the brain CBL (**a**,**d**,**g**), STR (**b**,**e**,**h**) and CTX (**c**,**f**,**i**). (**k**) Confusion matrices corresponding to each UMAP (**a**–**i**). The predicted and actual classification results for HD and WT astrocytes in the whole cell, cytoplasm, and nucleus for all three brain regions are listed in Table 1.

Spectral phenotyping will discriminate between WT and HD samples if their mean absorbance spectra differ. FTIR class is defined as disease (HD) or non-disease (WT). Thus, a robust disease prediction depends on the chemical features that contribute most to the differences (Fig. 1a). Because those features are not known a priori*,* we considered whether cell segmentation would identify a best subcellular site for spectral acquisition. For example, the high contrast of the nucleus is a desirable segment to extract discriminant IR or Raman spectral features^18,37,44,45^. However, if features of the cytosol provided a major contribution to the spectral differences, then the nuclear segment might not be ideal for disease predictions. The hyperspectral images were segmented (Fig. 4a–f) using the Otsu's algorithm^46^ (Fig. 4a,b) followed by the seed point-watershed algorithm (Fig. 4c–f)^47–50^. The cell segmentation was performed before the spectral preprocessing. Thus, the signatures from each segment were based on the integrated absorbance frequencies between 1670 and 1630 cm^−1^ (amide I band) for each pixel, and not on biochemical differences. Nonetheless, the (absorbance) difference between cytoplasm and condensed matter of the nucleus is large and the signatures derived from the whole cell, the cytoplasm and the nuclear segments were distinct in the WT and HD comparison (Fig. 5). The segmentation approach enabled a fast, semi-automated distinction between nuclear and cytoplasmic segments in the image relative to the whole cell (Fig. 4a–f). Pixels that were designated as nuclei (Fig. 4e) were estimated from the maximum intensity variation between the image background and foreground, where foreground is defined as the cell center and the background is the whole cell (Fig. 4b). The pixels, which were designated as the cytoplasm (Fig. 4f), were derived by subtracting the pixels designated as the nuclei (Fig. 4e) from those of the whole cell (Fig. 4d). The raw spectra from each segment were quality tested using our Python routine adapted from the Bruker OPUS software^51^. The test controlled for signal to noise ratio (SNR) and signal to water ratio (SWR) to allow selection of spectra that fit the robust criteria to be included in the spectral biomarker (Fig. 4g). The spectra were subsequently pre-processed to reduce other artifacts that occurred during the acquisition (Fig. 4h)^34,52^, as described in the methods section. Corrected spectra are displayed as second derivative curves throughout the results.

Indeed, in all cell segments, the mean absorbance spectra plotted as a second derivative, were different for WT and HD astrocytes (Fig. 4i–k, Supplementary Fig. 2), particularly in the lipid portion of the spectrum. For all three brain regions (CBL, STR and CTX), these differences are shown in the magnified views of spectra in then the 3050–2800 cm^−1^ region, originating from mainly lipids and the “fingerprint” (1800–900 cm^−1^) region (Fig. 4i–k, Supplementary Fig. 2). The “fingerprint” region comprises spectral features from lipids, but also contains features for proteins (amide bands), nucleic acids and carbohydrates (Fig. 1a). We tested whether cell segmentation mattered in the disease prediction. Each sample was classified by clustering the mean spectrum from each cell segment (either nucleus, cytoplasm, or whole cell). Class assignment was evaluated by clustering using either unsupervised Principal Component Analysis (PCA)^53^ (Supplementary Fig. 3a), or unsupervised non-linear Uniform Manifold Approximation and Projection (UMAP) method^54,55^ (Fig. 5a–i). While PCA assigns equal weights to all pairwise linear distances, UMAP is a non-linear method. Plots are unitless and reflect closest datapoints to define the clusters (Fig. 5a–i). Using either of these clustering techniques, biological classes were determined by the distance between the cluster centers, i.e., if samples are of a distinct class, the clusters will have little to no overlap. Indeed, the FTIR signature’s ability to distinguish control and disease states critically depended on the choice of the cell segment. For P2 astrocytes, the clustered spectra from disease or control astrocytes were well separated and predicted disease class in the three brain regions tested if the features were extracted from whole cells (Fig. 5a–c) or from cytoplasm segments (Fig. 5d–f), both of which contain the lipid-rich plasma membrane. In contrast, clusters from nuclear segments significantly overlapped and consistently worsened the prediction (Fig. 5g–i). This was obvious in both the UMAP (Fig. 5) and PCA (Supplementary Fig. 3) plots. PC loadings (Supplementary Fig. 3b) confirmed that sample (whole cells or cytoplasm segment) discrimination was based on lipid features (3050–2800 cm^−1^) and on spectral features in the “fingerprint region” lipid peaks (1740 cm^−1^, 1455 cm^−1^) and protein features at 1655 and 1535 cm^−1^ (amide I/II bands). Although changes to lipids are not unique to HD, their contribution to the disease signature in P2 astrocytes was significant. These molecules are not only vital to the health of the central nervous system, but lipids also are disrupted in Huntington’s disease^56,57^.

The quality of the classification was quantified in the PCA/UMAP analysis by a Silhouette score (S)^58^, which is a metric for how close each point in one cluster (cohesion) is to its neighboring clusters (separation) (Table 1). The metric is calculated on a − 1.0 to 1.0 scale with a higher score indicating datapoints that are closer to their own clusters than to other clusters. Indeed, the S for disease prediction (whole cell or cytoplasm) from all three brain regions ranged from 0.4 to greater than 0.7, indicating a good distinction between the two classes (Table 1). In contrast, the S for the nuclear segment ranged from 0.09 to 0.22 indicating that the control and disease signatures were not well-resolved. The spectral distinctions from the second derivative absorbance curves in all three regions are shown (Fig. 5). Shuffling and permutation^59^ of the FTIR datasets in each region confirmed that the classification was robust (*p* < 0.001) for cytoplasm and whole cell analysis (Table 1). UMAP, by its distance emphasis, was sensitive enough to reveal small differences among technical and biological replicates, which were not necessarily identified using PCA (Supplementary Fig. 4a). Nonetheless, using either approach, the disease prediction was robust (Fig. 5, Table 1, Supplementary Fig. 3, Supplementary Table 1) and reproducible in technical and biological preparations used throughout the analysis.

**Table 1:** Metrics for spectral classification (from Fig. 5).

	WT versus HD CBL			WT versus HD STR			WT versus HD CTX		
	NUC	CYT	CELL	NUC	CYT	CELL	NUC	CYT	CELL
S	0.09*	0.62*	0.78*	0.11*	0.39*	0.52*	0.22*	0.35*	0.41*
Sens	0.87	1.00	1.00	0.88	0.93	0.96	0.87	0.95	0.99
Spec	0.42	0.99	1.00	0.70	0.97	0.99	0.75	0.91	0.93
A	0.72	1.00	1.00	0.81	0.95	0.98	0.81	0.93	0.95

*S* silhouette score, *Sens* sensitivity, *Spec* specificity, *A* accuracy.

*p value < 0.001.

### Spectral phenotyping is accurate

The quality and accuracy of the classification was established from a confusion matrix (Fig. 5k) using a k-nearest neighbor (k_NN_) statistical model^60^. The confusion matrix is a signature classifier, which considers all data instances as either positive (disease) or negative (controls). The results of the confusion matrix for all three regions are shown and key statistical metrics are summarized (Fig. 5k). Indeed, the number of false positive and false negative assignments was consistently low, and accuracy (A) of correct assignment was over 90% for most samples using cytoplasmic or whole cell segments. The high sensitivity and specificity also indicated that a high proportion of disease or control samples were classified as such (Table 1). Thus, the disease prediction from unsupervised PCA (Supplementary Table 1) and UMAP was accurate.

**Figure 6:**
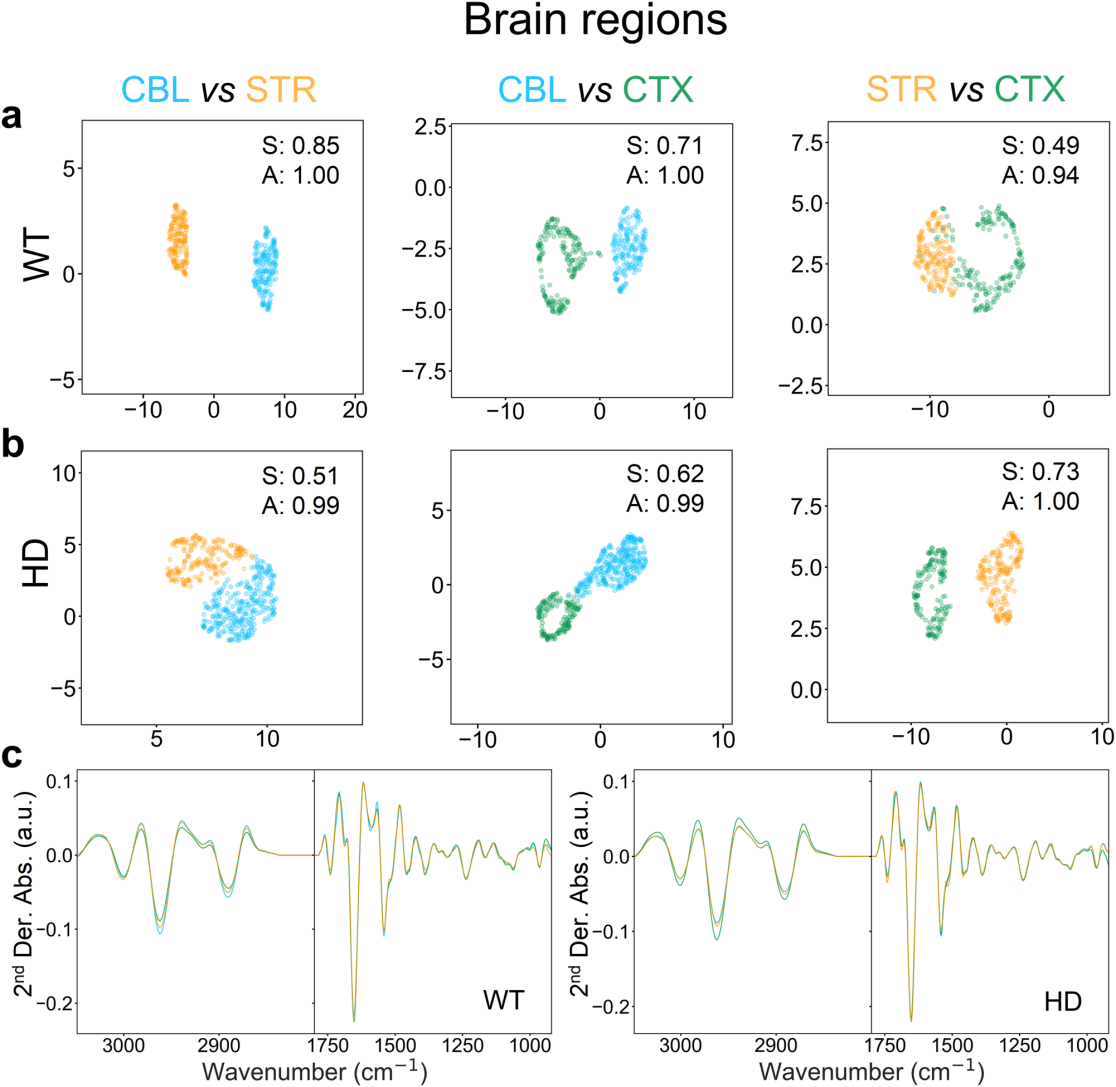
Astrocytes have regional signatures that are distinguishable by their FTIR signatures. (**a**,**b**) Pairwise classification of astrocytes isolated from the CBL, STR and CTX brain regions of SV40T WT (**a**) or HD (**b**) animals by UMAPs of 2^nd^ derivative normalized absorbance FTIR spectra (whole cells). (**c**) Average 2^nd^ derivative normalized spectra of WT (left) and HD (right) SV40T astrocytes from the CBL (blue), STR (orange), CTX (green) brain regions. Spectra are displayed between 3050 and 2800 cm^−1^ (lipid-rich region) and 1800–900 cm^−1^ (“fingerprint” region). S, silhouette score (*p* value < 0.001); A, accuracy.

We also evaluated whether the FTIR signature was sensitive enough to discriminate among astrocytes from distinct brain regions from either WT or HD animals (Fig. 6). This was a more stringent test of classification since the cells to be evaluated were of the same type (astrocytes) and shared the same genotype. The FTIR signature would differ only if the features reflected the spatial origins of the astrocytes. Surprisingly, the P2 astrocytes from WT mice as well as their HD littermates were characterized by a spatial identity as early as 2 days after birth (Fig. 6a,b). Thus, FTIR signatures recognized subtle differences (Fig. 6c) in the modifications among cellular molecules that defined their regional position. The FTIR signature predicted disease class in astrocytes at very early ages, consistent with growing evidence that HD is a developmental disorder^61^. The cluster separation among regions was good to excellent, with S ranging from around 0.4–0.85 depending on the regional comparison (Fig. 6a,b). Collectively, the results provided evidence that spectral phenotyping was able to predict disease class of astrocytes with high probability using a unique FTIR signature as the biomarker. Not only did it accurately predict disease class, but the FTIR signatures were able to discriminate between control and disease astrocytes, which were isolated as early as 2 days after birth and displayed no obvious phenotypic differences.

### The disease signatures are reproducible

**Figure 7:**
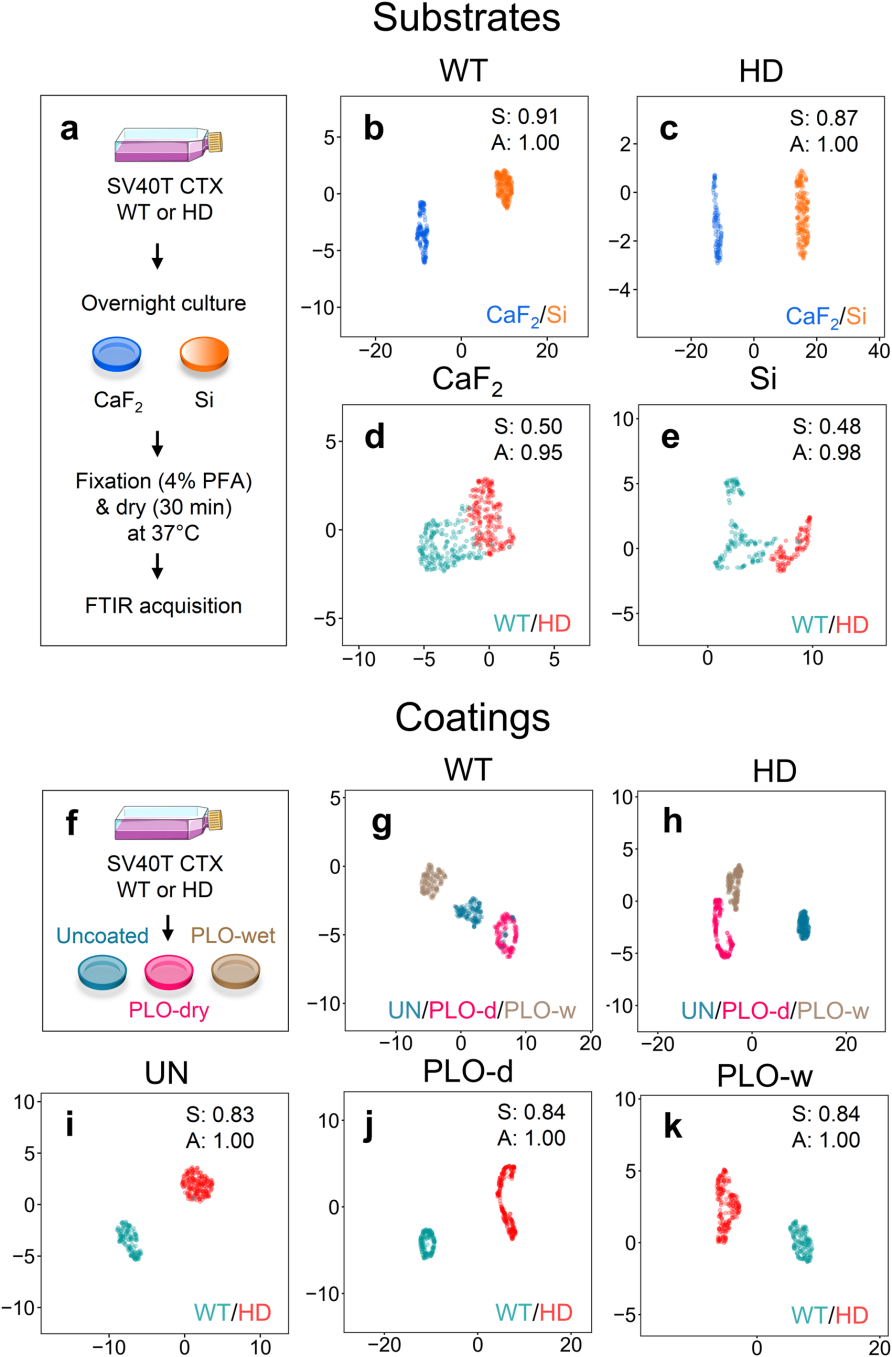
FTIR substrates and coatings have an influence on cell spectra without altering disease/control classification. (**a**) Experimental protocol schematic representing SV40T CTX WT or HD astrocytes cultured overnight on CaF_2_ and Si substrates. Cells were fixed and dried prior to the FTIR acquisition. (**b**,**c**) UMAP clustering results of WT (**b**) or HD (**c**) cells grown on CaF_2_ and Si substrates. (**d**,**e**) UMAP classification of WT and HD astrocytes grown on either CaF_2_ (**d**) or Si (**e**) substrates. (**f**) Schematic of substrate coating effect experiment following the same procedure as in (**a**). SV40T CTX WT or HD astrocytes were cultured overnight onto CaF_2_ substrates uncoated (UN), with poly-L-ornithine dry (PLO-d) or poly-L-ornithine wet (PLO-w) coatings. (**g**,**h**) UMAP clustering results for all three coatings on CaF_2_ substrates for WT (**g**) or HD (**h**) cells. (**i-k**) UMAP classification of WT and HD astrocytes grown on CaF_2_ substrates uncoated (**i**) or coated with PLO-d (**j**) and PLO-w (**k**). All UMAP analyses were performed on 2^nd^ derivative normalized absorbance FTIR spectra of whole cells. S, silhouette score (*p* value < 0.001); A, accuracy.

The astrocytes samples were isolated from distinct litters of pups and the slides generated were stored before spectral measurements. To ensure that the FTIR classification was robust, we measured the reproducibility of the FTIR signature for cell preparations under relevant condition of temperature, storage, and slide preparation. We evaluated the impact of slide substrate type (Fig. 7a–e), slide coating (Fig. 7f–k), sample storage time and storage temperature (Supplementary Fig. 4) on the accuracy of the FTIR disease prediction. FTIR spectra were acquired using transmission mode, which requires IR light to pass through the slide and sample. Calcium fluoride (CaF_2_) or silicon (Si) are typical substrates for this purpose (Fig. 7a). In our experiments, CaF_2_ was used most often. Although the choice of substrate had an impact on the resulting FTIR signature (Fig. 7b,c), WT and HD discrimination was successful using spectral phenotyping as long as samples were measured and compared using the same substrate (Fig. 7d,e). The predictions had a good S and high A (Fig. 7d,e). Slide coatings are not always needed but are often used to improve cell adherence to the substrate. A common coating is poly-L-ornithine (PLO), which is used wet (PLO-w) or dry (PLO-d) in various preparation protocols. Samples were prepared as in Fig. 7a, and we tested whether cells layered onto wet or dry PLO coating altered the disease prediction relative to uncoated slides (Fig. 7f). Although the slide coatings themselves had an impact on the resulting FTIR signature (Fig. 7g,h), WT and HD discrimination was successful independent of coating, as long as the compared samples were measured under the same conditions (Fig. 7i–k).

We also defined the impact of sample storage on the robustness of the disease prediction. Slides were prepared and stored at RT (Supplementary Fig. 4b) or − 80 °C (Supplementary Fig. 4c), for various periods from which the FTIR signature was measured before and after storage. Sample storage at room temperature (RT) yielded a relatively low S indicating significant overlap after a day of storage up to 2 weeks (Supplementary Fig. 4b). The spectral signatures were not reproducible during long term storage (5 months) at − 80 °C (i.e., class separation) (Supplementary Fig. 4c). However, signatures were stable for at least 2 weeks if samples measured at RT were returned to storage at − 80 °C between subsequent measurements (freeze–thaw) (Supplementary Fig. 4d). Although there are inevitable chemical changes that occur when cells are fixed, as previously reported^38,40,62,63^, fixation did not impair disease classification as long as both samples were fixed under the same conditions. Collectively, these results defined conditions for sample preparation that resulted in robust measurements.

### FTIR phenotyping is a general use tool for disease prediction in human cells

**Figure 8:**
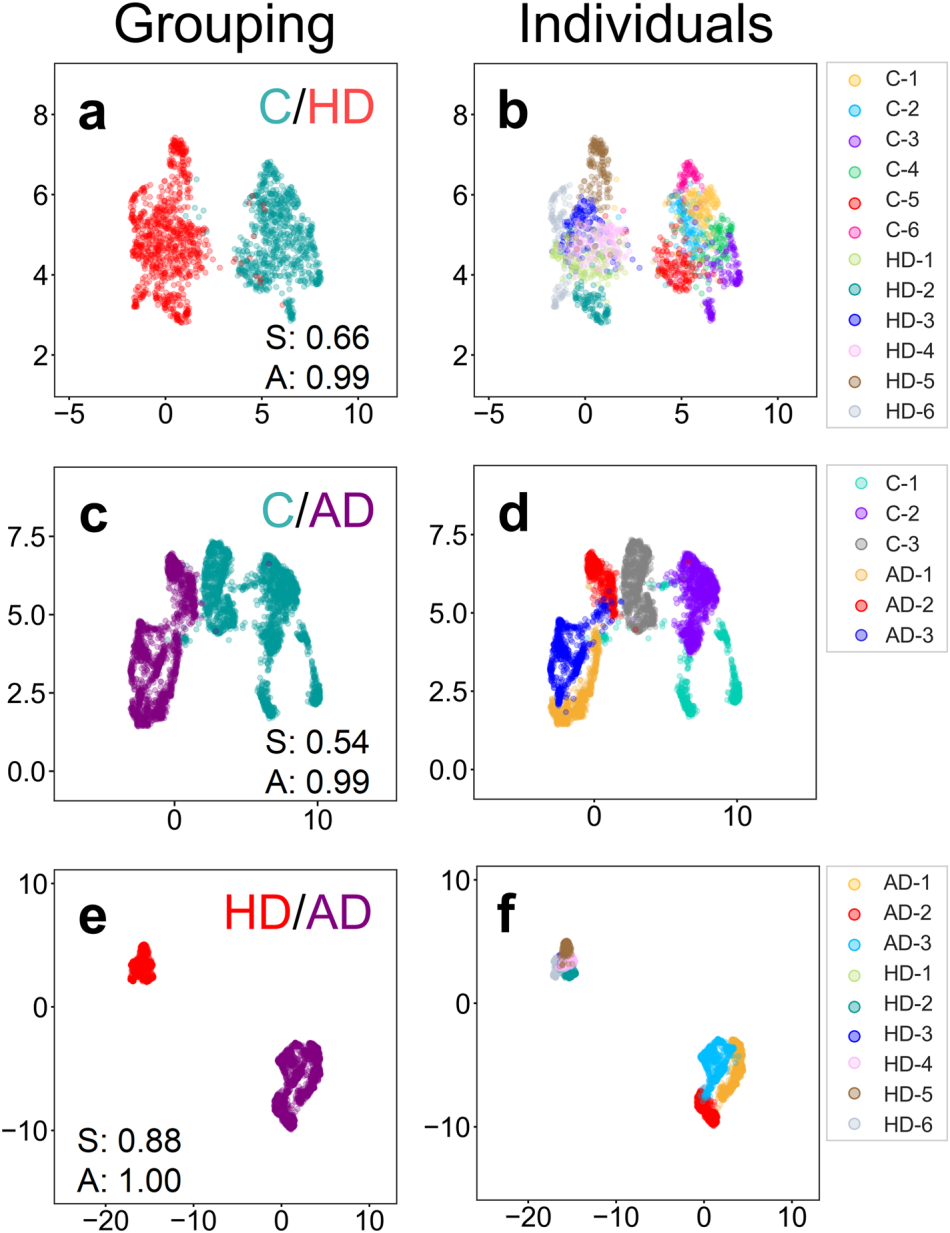
Spectral phenotyping can predict human neurodegenerative disease class from fibroblasts. FTIR spectra from human skin fibroblasts of controls (C) *versus* Huntington’s disease (HD, **a**,**b**), Alzheimer’s disease (AD, **c**,**d**) or a comparison of HD and AD (**e**,**f**) were evaluated by UMAP. The UMAP plots are the results of either pooled control or pooled disease samples (**a**,**c**,**e**), or displayed per individuals (**b**,**d**,**f**). All UMAP analyses were performed on 2^nd^ derivative normalized FTIR spectra of whole cells. S, silhouette score (*p* value < 0.001); A, accuracy.

In practice, the usefulness of FTIR spectral phenotyping as a biomarker is its ability to accurately classify human disease cells. Since the brain is not accessible for analysis, we considered whether HD patient fibroblasts might be used as surrogates. The premise being that these cells shared the same genotype with HD brain cells and might undergo chemical changes that tracked with disease. HD human fibroblast samples were obtained from the Coriell repository. The demographics of each patient are listed (Supplementary Table 2). Spectral phenotyping was evaluated as a classifier by evaluating either pooled samples (Fig. 8a) or as individual samples (Fig. 8b). PCA (Supplementary Fig. 5) or UMAP (Fig. 8) clustering was used to determine the disease class.

All samples were gender matched (male). For HD, most of the control and patients were of similar age (around 60 years), but two HD patients were younger (around 35 years) than controls and one control was older (78 years) than the HD patients. Despite the age variations, the disease classification, as judged by either UMAP (Fig. 8a) or PCA (Supplementary Fig. 5a), was robust for human HD fibroblasts, with an S of 0.66 and high A of 0.99 (Fig. 8a). Mean spectra for control and HD fibroblasts are displayed (Supplementary Fig. 6). The results suggested that there were at least some chemical features that are shared among HD patients, which were distinct from those of controls. Although individual HD patients and controls often formed their own clusters (Fig. 8b), these samples grouped within larger clusters according to disease class (Fig. 8a,b). Thus, the human HD fibroblast results added significance to spectral phenotyping since it was effective in classifying HD class across species, i.e., mouse (Fig. 5) or human (Fig. 8a) harboring the mutant disease gene. Although we expected more variation among human samples relative to those previously measured in the mouse samples (Fig. 5), the chemical biomarker for HD cells distinguished disease class regardless of species or cell type, i.e., the predictions for human fibroblasts (Fig. 8a) and mouse astrocytes (Fig. 5) were equally robust.

The accuracy of disease classification using the FTIR biomarker was not limited to HD. Three AD human samples were also classified relative to age and gender matched controls. All male AD patients were between 60 and 66 years as compared to the male controls which ranged from 60 to 78 years. Like the HD results, all three AD patient samples clustered as a group that was distinct from controls even though the underlying mutations were unknown for any sample (Fig. 8c). As with HD, individual control and AD patients were resolvable from each other (Fig. 8d) as judged by either PCA (Supplementary Fig. 5d) or UMAP (Fig. 8d), but overall, the samples grouped according to their disease class, validating the disease prediction potential of fibroblasts. HD and AD are late onset diseases but differ significantly in that the first is due to a dominant and fatal genetic disorder, while in the latter the underlying mutation is unknown for most patients and death does not always occur from the disease. Yet, robust classification of human fibroblasts from each of these neurodegenerative diseases was possible even in what visually appeared to be homogeneous and indistinguishable cultures. Thus, the unique FTIR chemical biomarker was accurate in predicting disease class in cells of different species, of distinct types, and between two neurodegenerative diseases.

## Discussion

Cells have chemical features that set them apart, but those features can be subtle and difficult to detect. However, these subtle chemical differences are identified by FTIR spectral phenotyping. Here we show that the spectral imaging approach reproducibly and reliably predicts control or disease classification using an FTIR signature as a biomarker. Not only did it accurately predict disease class, but the FTIR signatures were able to discriminate between control and disease astrocytes from animals as early as 2 days after birth. At this stage, WT and HD animals have distinct genotypes, but the number of neurons, morphology and antibody staining patterns in the brain are equivalent. In the absence of obvious pathology, FTIR signatures correctly classed them as control or disease. We propose that spectral phenotyping will provide a mechanism to detect and track even subtle changes in a cell’s chemical states with high probability at early stages of disease progression. Classification by FTIR is possible using standard FTIR equipment which is available for use in universities and in hospital environments. The FTIR signature is robust and applies across disease types, cell types, and species in these proof of principle experiments. We predict that spectral phenotyping can be developed to broadly identify cellular changes of state such as those that occur in disease, viral infection^64^, drug exposure, and embryonic development, which will be tested in future studies.

More than a decade of ground-breaking work has catapulted FTIR imaging as a powerful new tool with great promise for clinical applications^24,25,35,65^. Recent technological advances have and will continue to improve the technique. For example, synchrotron radiation is 100–1000 times brighter than conventional thermal IR light, providing a better spatial resolution and leading to unprecedented chemical probing of live cells^39^. The use of Quantum Cascade Lasers (QCLs) offers the ability to scan specific wavenumbers of interest, which decrease the time of acquisition^66,67^. Submicrometer spatial resolution is now achievable by mid-IR photothermal microspectroscopy with commercially available bench top instruments. This method has been used to observe amyloid protein aggregates at subcellular level, along the neurites and dendritic spines of neurons^68^. Thus, FTIR spectroscopy is increasing in its capabilities to classify both fixed^38,40,62,63^ and live cells^39^.

With respect to clinical application, our spectral phenotyping method offers three potential advances. First, we show here that spectral phenotyping can accurately classify disease states before manifest symptoms. If disease pathology is well understood, FTIR spectroscopy is not needed to classify post-mortem tissue at the end of life. As an early biomarker, however, spectral phenotyping would be invaluable in disease predictions for asymptomatic patients during life or for the many diseases where a diagnosis is difficult or unclear. As an example, in the absence of cognitive decline, a diagnosis of a pre-symptomatic AD patient is tentative and disease candidates are determined based on low levels of amyloid-beta peptide in the blood or in MRI brain images. Yet, a diagnosis is uncertain since these aggregates are also present in the normal aging population. Similarly, infrared spectroscopy has been useful in examining the conformation and structure of mature aggregates^69–74^, but they are not present in HD fibroblasts or in HD mouse models at early stages of disease. Second, using segmentation and UMAP analysis, robust disease predictions were achievable. UMAP, unlike PCA, is a non-linear dimension reduction method. UMAP prioritizes distances, i.e., the closeness of neighbors, and maximizes the separation among samples, allowing robust clustering for a larger number of samples. Although whole cells or nuclei have been common regions for feature extraction by us and others, we find that subcellular segmentation is an essential part of the analysis algorithm since misclassification can occur if the correct segments are not used. Third, each signature comprises hundreds of cells allowing a robust signature and the analysis is relatively rapid and economical. With data in hand for segmentation, the processing time of 16,384 spectra contained in one FOV on our local computer is around 160 ms. The entire acquisition time for hundreds of cells, required for robust classification, is most often complete in under an hour with an FPA detector, and off-line analysis is complete in two hours. High throughput is possible using an assembly line approach. Moreover, we expect that the speed of FTIR imaging will improve further with technological advances, and that the use of IR spectral signatures will increase throughput and will outpace other approaches as a basis for accurate disease classification.

The importance of a biomarker for disease predictions cannot be overstated. There is a desperate need to develop therapeutic compounds, but we have no classification criteria by which to judge when to start or stop treatment, which is also costly and time consuming. Thus, the gap between the incidence of disease and our ability to treat patients is growing exponentially. Although the use of serum samples in FTIR analysis has advanced considerably^22,23^, we show here that surrogate skin cells at early ages can be used for reliable disease predictions of neurological and neurodegenerative diseases. Although they have distinct functions from that of neurons, peripheral cells such as fibroblasts are stable, maintain the genetic background of the patient^75^, and the chemical alterations which track with disease are detectable by FTIR spectroscopy. The wide availability of lymphoblasts, fibroblasts, and induced pluripotent stem cells (iPSCs) provide new opportunities to collect samples from living patients with neurological disorders, and track disease endpoints at very early biological states with minimum discomfort. A biomarker which is sensitive to disease progression and its reversal would be valuable in that early detection would lower the cost and time of treatment by predicting a treatment window.

There will be solvable challenges. In these proof of principle experiments, we have tested spectral phenotyping on a small number of human cell lines. Disease prediction has high accuracy in the age and gender matched samples and controls used in this study. These results suggest that spectral phenotyping holds promise as a clinically relevant biological tool. However, as we move towards larger populations, it is likely that factors such as lifestyle, ethnicity and medical background will introduce more variability. More extensive analysis using additional statistical or clinical parameters may be necessary to retain a robust disease prediction by FTIR spectroscopy. Nonetheless, classification using FTIR signatures is accurate, and the measurements require minimal sample preparation and no a priori knowledge of the sample, which can be highly useful for unbiased disease classification (i.e., disease *versus* non-disease). Currently, we do not know if diagnosis of a disease state by FTIR spectroscopy requires a comparison with a control sample or whether each disease has its own signature. However, signature specificity will be an important consideration. In theory, millions of combinatorial signatures are possible in the mid-IR range between 4000 and 800 cm^−1^. However, there will be overlap and redundancy among spectral features, placing limits to the number of discrete signatures, a possibility that will require more extensive analysis. For example, lipids can change in many disorders or abnormalities, and therefore are not specific to a particular disease. Although the limits of signature “uniqueness” remains to be determined, our initial results are promising. The patients we have analyzed from the Coriell repository are unrelated and therefore, are unlikely to have shared the same lifestyle, have the same cholesterol levels, or the same diet, but disease predictions for both HD and AD populations are robust when compared to controls. Although both diseases are associated with lipid abnormalities and both have lipid features that contribute significantly to their signature spectra, when compared to each other, AD and HD form distinct groups that do not overlap (Fig. 8).

In short, spectral phenotyping by FTIR spectroscopy meets the ever-increasing demand to measure unperturbed, native states, with wide ranging applications in cell biology, diagnoses, and predictive biology**.** The approach positions us in unprecedented ways to predict cells that are diseased or behave differently with age, type or during disease progression, all of which have been difficult to achieve reliably using other methods.

## Methods

### Animals and cell lines

Breeding and use of *HhdQ(150/150)* and *C57Bl6J* mice was performed as reported previously^42,43^. All procedures involving animals were approved by the Lawrence Berkeley National Laboratory Animal Welfare and Research Committee and performed in accordance with the relevant guidelines and regulations. The use of live animal was carried out in compliance with the ARRIVE guidelines. Established human cell lines used in this study include AD and HD human fibroblasts obtained from the Coriell Institute repository. The demographics and phenotypic data are reported for each cell line in Supplementary Table 2.

### Dissections and isolation of primary astrocyte cultures

Mouse primary astrocytes were isolated from various brain regions as previously described^42,43^. Briefly, intact brains were collected from postnatal day 1–3 pups (called P2) for either genotype (*HhdQ(150/150)* or *C57Bl6J* mice). Brain regions (cerebellum, striatum and cortex) were isolated in a solution of Phosphate Buffer Saline (PBS) on ice. The regions of 4–7 pups of each genotype were pooled and digested in 10 mL 0.25% Trypsin-Ethylenediaminetetraacetic acid (EDTA) (Gibco 25300056) in PBS for 15 min at 37˚C. Tissue pieces were pelleted (5 min, 300 rcf, room temperature (RT)) and then gently triturated 20–30 times in pre-warmed potent media (DMEM (Gibco 10569044), 20% FBS (JRS 43635), 2.5 mM glucose, 2 mM sodium pyruvate, 2 mM glutamax, 1 × non-essential amino acids (Quality Biologicals 116-078-721EA), and 1 × antibiotic/antimycotic (Gibco #15240062) using a 5 mL pipet, to dissociate into single cells. Each cell suspension was plated into poly-l-ornithine (VWR 103,701–204) coated T75 culture flasks and cultured for 7–10 days (at 37 °C, 5% CO_2_), with media exchanges every 2–3 days. Cells were re-passaged twice to enrich for astrocytes. Astrocyte cell purity and homogeneity was tested by immunofluorescent analysis using anti-Glial Fibrillary Acidic Protein (GLAST) antibody (Invitrogen SPM498).

### SV40T immortalized astrocyte cultures

Primary cells were transformed with SV40 Large T antigen (ABM LV660), according to the manufacturer’s protocol, to create clonally derived immortalized cell lines. Briefly, logarithmically growing primary astrocytes in 6 well dishes with 1 mL potent media, were treated with 1 × 10^6^ units of high-titer SV40T lentiviral stock (ABM LV660), 5 µg/mL polybrene (EMD Millipore TR-1003-G) and 20 µL of ViralPlus Transduction Enhancer (ABM G698). Following 1 day of culture, cells were washed with fresh media and allowed to grow for an additional 3 days. Cells were then replated into two 10 cm diameter dishes and cultured for 4–6 days with 0.1 µg/mL puromycin. Individual clones were selected using cloning discs (Sigma Z374431) and grown up individually.

### Immunocytochemistry

Cells were fixed in freshly prepared 4% paraformaldehyde (PFA) (10 min at RT in the dark), then incubated with 100 mM Glycine, 0.1% Triton X-100, 0.05% Tween-20 in PBS (5 min), and blocked (1–2 h) in blocking solution (PBS, 3% Bovine Serum Albumin (BSA), 3% goat serum, 3% donkey serum, 0.03% triton X-100). Primary antibody [1:500 rabbit anti-GLAST (Invitrogen SPM498)] diluted in 10% blocking solution/PBS was added for 1 h, followed by 3 washes with PBS (5 min each). Appropriate secondary antibody [1:1000 donkey anti-rabbit Alexa 546 (Invitrogen A10040)], diluted in 10% blocking solution/PBS was then applied along with 0.5 µM DAPI (30 min) for nuclear staining followed by 2 washes in PBS. Slides were coated with Aqua Polymount (Fisher Scientific NC9439247), covered by a #1.5 coverslip, sealed with clear nailpolish and stored (− 20 °C). Slides were imaged using a Zeiss 710 confocal microscope at 1 A.U., using either 20 ×  (0.8 N/A)/air or 63 × (1.4 N/A)/oil lenses.

### Grip test

For the grip strength-endurance test, mice were lowered onto a parallel rod (diameter < 0.25 cm) placed 50 cm above a padded surface. The mice were allowed to grab the rod with their forelimbs, after which they were released and scored for length of time they could hold onto the bar (maximum 30 s). Mice were tested consecutively 3 times at each age. The maximum length of time they were able to hold on was recorded for analysis.

### MitoTracker Cell Staining

Staining was done according to the manufacturer’s instructions. Briefly, astrocyte cells were plated and allowed to grow in growth media until they reached 60–70% confluence. Media was removed and replaced with fresh media containing 100 nM Mitotracker Green FM. Cells were incubated for 30 min at 37 °C and 5% CO_2_ after which the media was removed, cells were washed with PBS and later fixed with 4% PFA containing 300 nM DAPI for 15 min. Cells were then re-washed with PBS and imaged.

### Western blot analysis

Astrocytes were plated on 10 cm cell culture plates in growth medium, transfected, and allowed to express heterologous proteins (16–20 h). Cells were then gently washed with ice cold PBS (pH 7.4) and scraped off in lysis buffer (200 μl RIPA buffer (ThermoFisher#89900) supplemented with HALT protease inhibitor (ThermoFisher#7842) and 5 µg/mL DNase I (ThermoFisher# 18047-019), triturated (20× with 200 µL pipet) and sonicated (3 × 15 s on ice). Protein concentration was determined using Pierce 660 nm protein assay kit (ThemoFisher# 22662) and relevant protein amounts (5–15 μg) were brought up in NuPage LDS sample buffer (ThermoFisher#NP0007) and NuPage sample reducing agent (ThermoFisher#NP0004). Samples were heated at 95 °C for 10 min and debris was pelleted (20,000 rcf, 10 min, room temperature (RT)). Samples were resolved on either 4–12%, 8–16% or 4–10% Novex Tris–Glycine SDS-Page mini gels (ThermoFisher) in Novex Tris–Glycine SDS running buffer at RT and transferred onto nitrocellulose membranes (0.2 μm) using BioRad Trans-blot Turbo transfer system (according to manufacturer’s protocol). Blots were washed with PBST (pH 7.4), general protein visualized using Ponceau S (SigmaAldrich#P7170), then rewashed with PBST. Blots were blocked in blocking buffer (5% Non-Fat Dry Milk (NFDM) in PBST (pH 7.4)) then probed with primary antibody (1:10,000 in blocking buffer) in a sealed pouch, with rocking for 1 h at RT. Blots were washed (3×) 10 min using PBST with rocking, and probed with secondary HRP labelled antibody (1:15,000 in blocking buffer) in a sealed pouch, with rocking for 30 min at RT prior to final washes (3×) 10 min using PBST. HRP was visualized using either the ECL Prime or ECL select chemiluminescent detection kits (SigmaAldrich) according to manufacturer’s protocols and imaged on a BioRad VersaDoc imaging system. Primary antibodies used were mouse anti-Htt (Millipore #MAB-2166)(htt), mouse anti-polyQ (DSHB #MW1)(mhtt), goat anti-GAPDH (Genscript #A00191). The secondary antibodies were goat anti-mouse HRP conjugate (Thermo Fisher Sci #G21040) and rabbit anti-goat HRP conjugate (Thermo Fisher Sci #31402).

### Sample preparation for spectral analysis

Dissociated cells in potent media were plated onto IR sterile substrates (25 mm × 1 mm calcium fluoride (CaF_2_) or silicon (Si) windows (Crystran Ltd, UK) inside wells of a 6 well plate. Substrates were either uncoated or coated with poly-l-ornithine (VWR 103701-204). ‘Wet’ coating involved incubating the substrates with 0.01% poly-l-ornithine for 30 min at room temperature (RT) and washing twice with PBS. ‘Dry’ coating involved incubation with poly-l-ornithine, removal of the solution by pipet and allowing the substrate to dry inside a laminar flow hood. Cells were grown 1–2 days (at 37 °C, 5% CO_2_). The media was removed, and slides were rinsed twice with PBS before cell fixation with 4% PFA in PBS for 10 min. Following fixation, the slides were rinsed with ultra-pure water (MilliQ water). The washed cells were dried at 37 °C for 30 min and kept in dark boxes with desiccants at either RT or in an − 80 °C freezer prior to spectral analysis.

### The methodology for spectral phenotyping

#### FTIR spectral imaging acquisitions

FTIR spectral images were collected using an Agilent Cary 670 FTIR spectrometer coupled to an Agilent Cary 620 FTIR microscope (Agilent Technologies, USA) with a 128 by 128 pixel liquid nitrogen cooled Mercury Cadmium Telluride (MCT) Focal Plane Array (FPA) detector. The Agilent system is also equipped with an in-built purging system allowing the maintenance of a low relative humidity during acquisitions. Images were obtained from multiple tiles of 704 μm by 704 µm acquired with a 15× magnification objective and condenser resulting in a projected pixel size of 5.5 µm. Spectral data were collected using the Agilent Resolutions Pro software in the transmission mode, by the co-addition of 256 and 128 scans for the background and samples respectively, at a spectral resolution of 4 cm^−1^ over the spectral range 4000–800 cm^−1^.

#### Segmentation

All spectral data were processed using our software written in Python 3. We used the Otsu's threshold algorithm^46^ to delineate subcellular segments for the spectral analysis. Otsu's algorithm is a semi-automated thresholding approach to define foreground and background in a grayscale image^46,76^. Since hyperspectral images were acquired, they were reduced into high contrast 2D images based on the integrated absorbance frequencies between 1670 and 1630 cm^−1^ (amide I band) for each pixel (Fig. 1b). For two classes, (e.g., foreground and background) the optimal threshold is chosen when Otsu's algorithm has maximized the inter-class variation. For all types of cells, we used a modified Otsu's algorithm which allows for local thresholding of 2D images, by applying the same principle, but on user-defined (size and shape) disk shaped pixel blocks. This "dynamic thresholding" approach is useful when the background of the image is non-uniform. Then, individual cells and cell nuclei were defined using the seed-watershed algorithm for separating different objects in an image^47–50,76^. The locations of nuclei centers were used as "seed points" in the watershed method, which is a topographic distance algorithm^47,48^. From these seed points, "basins" are flooded and separated by "watershed" lines when they meet. These watershed lines correspond to the estimated edges of the basins. In our case, this step is used to estimate the pixels of entire cells and cell nuclei. The cytoplasm pixels were derived by subtracting the designated nucleus pixels from those of the whole cell. Attributed nucleus and cytoplasm pixels were eroded by two pixels to enhance cytoplasm and nucleus or cell–cell delineation. Finally, a mean spectrum was computed from each cell segment.

#### Quality testing

A quality test was applied to each spectrum using our routine adapted from the commercially available Bruker OPUS software^51^. Extracted spectra were quality tested to control for absorbance (A), signal to noise ratio (SNR), and signal to water vapor ratio (SWR). In our case, the cutoff value for each parameter was calculated based on 3332 spectra (nuclei and cytoplasm) coming from 1666 fixed, cultured astrocytes. The lower and higher bound values for A were chosen arbitrarily to the mean absorbance ± 5 standard deviations. SNR was calculated from parameters S1 and S2 corresponding to the difference between the minimum and maximum value of the first derivative on the band 1600–1700 cm^−1^ (amide I) and 960–1260 cm^−1^ (sugar-ring), divided by the noise (N) intensity over the 2100–2000 cm^−1^ region, where no absorbance is typically present in biological samples. Spectra were rejected when S1/N and S2/N were equal to the mean value of these equations ± 1 standard deviation. SWR was calculated from S1, S2 divided by the water vapor content (WVC) parameter which is the difference between the maximum and minimum value of the first derivative calculated between the 1847–1837 cm^−1^ range, which exhibits a strong water vapor absorbance and no sample contribution. Spectra were rejected when S1/WVC and S2/WVC were equal to the mean value of these equations ± 1 standard deviation. Using these cutoff values, 80% of the 3332 spectra passed the quality test.

#### Pre-processing

To extract the chemical information embedded in the absorbance values of the spectra, we applied a previously reported technique to minimize physical artifacts that might have occurred during the acquisition^34,52^. Initially raw spectra which passed the quality test were cut and pre-processed over the 4000–900 cm^−1^ range. Spectra were smoothed using the Savitzky–Golay method^77^ before applying a second derivative (21 points, 2nd polynomial order) for baseline correction and spectral contrast optimization. Then, spectra were vector normalized to enable their comparison^52^*.* To simplify spectral feature visualization, the pre-processed mean spectra were displayed between the lipid-rich (3050–2800 cm^−1^) and the “fingerprint” (1800–900 cm^−1^) regions.

#### Biological classification and statistics

Biological classification was accomplished by clustering the data after dimensionality reduction using UMAP^54,55^ or PCA^53^. The separation of the data into clusters indicates the different biological classes. PCA maximizes the linear (Euclidean distance) variance between spectra projected in 2D while UMAP is a topological method that optimizes the connectedness of spectra in the dataset. The quality of the clusters is defined by a Silhouette score (S)^58^, which is computed based on the mean intra-cluster distance (the distance between one cell and all others in the same cluster) and the mean distance between one cell and all other cells of the next nearest cluster (mean nearest-cluster distance). Individual spectra were classified using a k-nearest neighbor (k_NN_) statistical model^60^ and accuracy was calculated from a confusion matrix^78^ (Fig. 5k, Table 1). In a k_NN_ model, the k training points that are nearest to each test datapoint are considered, and the predicted identity is the most commonly occurring label among those k points. The analysis was performed with k-fold cross validation with k = 3. This means that each dataset was randomly shuffled and evenly split into 3 subsets. A k_NN_ model was trained on two of these subsets and evaluated on the third. As a final step, each of the subsets were merged and the datapoints were grouped according to control or disease and the number of correct and incorrect assignments was calculated. Thus, the confusion matrix summarizes the performance of the classifier, by considering all datapoints as either positive (disease) or negative (controls). A true positive (TP) is a sample which is correctly classified as HD (disease). A true negative (TN) refers to the samples without the mutant gene, which are correctly assigned as a WT (control). False positives (FP) are spectra from a control sample, which are incorrectly identified as a disease sample. A false negative (FN) is a disease sample, which is incorrectly classified as a control cell. Using these parameters, the accuracy (A) (Eq. 1), specificity (SPEC) (Eq. 2), and sensitivity (SEN) (Eq. 3) were derived, respectively. Each parameter is scored from best (1.0) to worst (0).

1 $${\text{A}} = \left( {{\text{TP}} + {\text{TN}}} \right)/\left( {{\text{TP}} + {\text{TN}} + {\text{FP}} + {\text{FN}}} \right);\;{\text{the number of correct assignments/total number of samples}}$$

2 $${\text{SPEC}} = {\text{TN}}/\left( {{\text{TN}} + {\text{FP}}} \right);{\text{ is a true negative rate}}$$

3 $${\text{SEN}} = {\text{TP}}/\left( {{\text{TP}} + {\text{FN}}} \right);{\text{ is true positive rate}}$$

## Supplementary Information

Supplementary Information.

## Data Availability

Data available upon request from the authors.
