# Supplementary material for: An infrared spectral biomarker accurately predicts neurodegenerative disease class in the absence of overt symptoms
Source: Sci Rep. 2021 Aug 2;11:15598. doi: 10.1038/s41598-021-93686-8 (PMC8329289; doi:10.1038/s41598-021-93686-8)
Supplement: Supplementary file 1 — Supplementary Information. [file 41598_2021_93686_MOESM1_ESM.pdf]

Supplementary Information

An infrared spectral biomarker accurately predicts neurodegenerative disease class in the absence of overt symptoms

Lila Lovergne[1#], Dhruba Ghosh[2#], Renaud Schuck[1#], Aris A. Polyzos[1#], Andrew D. Chen[2], Michael C. Martin[3], Edward S. Barnard[4], James B. Brown[2,5] and Cynthia T. McMurray[1*]

[1]Division of Molecular Biophysics and Integrated Bioimaging, Lawrence Berkeley National Laboratory, Berkeley, CA 94720.

[2]Department of Statistics, University of California Berkeley, Berkeley, CA 94720.

[3]Advanced Light Source, Lawrence Berkeley National Laboratory, Berkeley, CA 94720.

[4]Molecular Foundry, Lawrence Berkeley National Laboratory, Berkeley, CA 94720.

[5]Division of Environmental Genomics and Systems Biology, Lawrence Berkeley National Laboratory, Berkeley, CA 94720.

[#]These authors contributed equally

*To whom correspondence should be addressed:

Tel: (510) 486-6526; Fax: (510) 486-6880

Email: ctmcmurray@lbl.gov

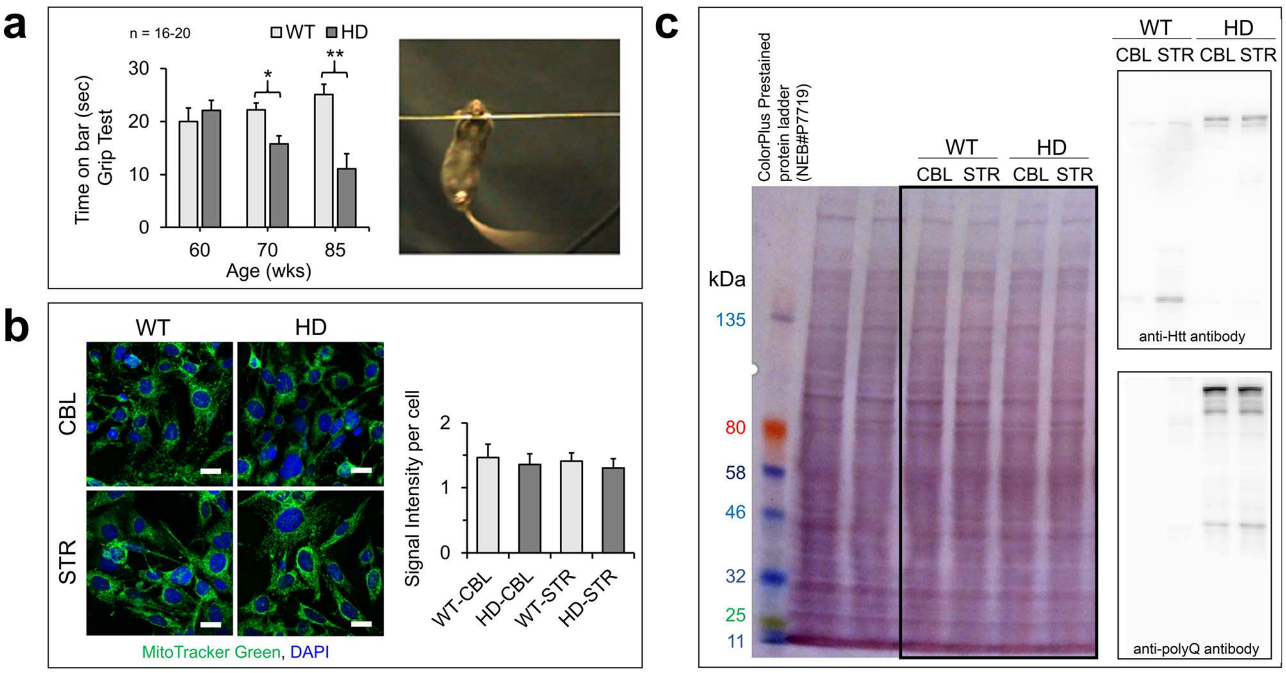

Supplementary Figure 1. (a) Grip test for motor function. The time in seconds is a measure of duration for gripping the bar (right). Performance is plotted as time (sec) *versus* age (wks) in WT and HD animals (left). The WT and HD animals have similar grip performance up to 60 weeks. n= 16; * *p*-value: < 0.05; ** *p*-value: < 0.005 (Student's *t*-test, 2 tailed, equal variance homoscedastic). **(b)** (left) Fluorescence staining of astrocytes with Mitotracker Green (green) to visualize mitochondria number and activity, which are equivalent in WT and HD cells. DAPI staining (blue) indicates the position of the nucleus. To the right is quantification of mitochondrial staining in astrocyte cultures from the CBL or the STR, as indicated. Light gray is WT and dark gray is HD; n= 50 (right). Variance is reported as standard error. The scale bar is 10 µm. **(c)** Full length uncropped western gels of normal and mutant huntingtin protein corresponding to the cropped images in Fig. 3f. (Left) Total protein loading control for the WT and HD animals in the cerebellum (CBL) and striatum (STR), as indicated, visualized with No-Stain Protein Labelling Reagent (Thermofisher). The boxed region corresponds to the four lanes in the gels on the right. (Right) The nitrocellulose blots were probed with an anti-htt antibody (upper blot), to the normal huntingtin protein in the WT or to the faster migrating band in the heterozygous HD sample. The anti-polyQ antibody (lower blot) primarily detects the mutant protein in the slower migrating band in the HD sample.

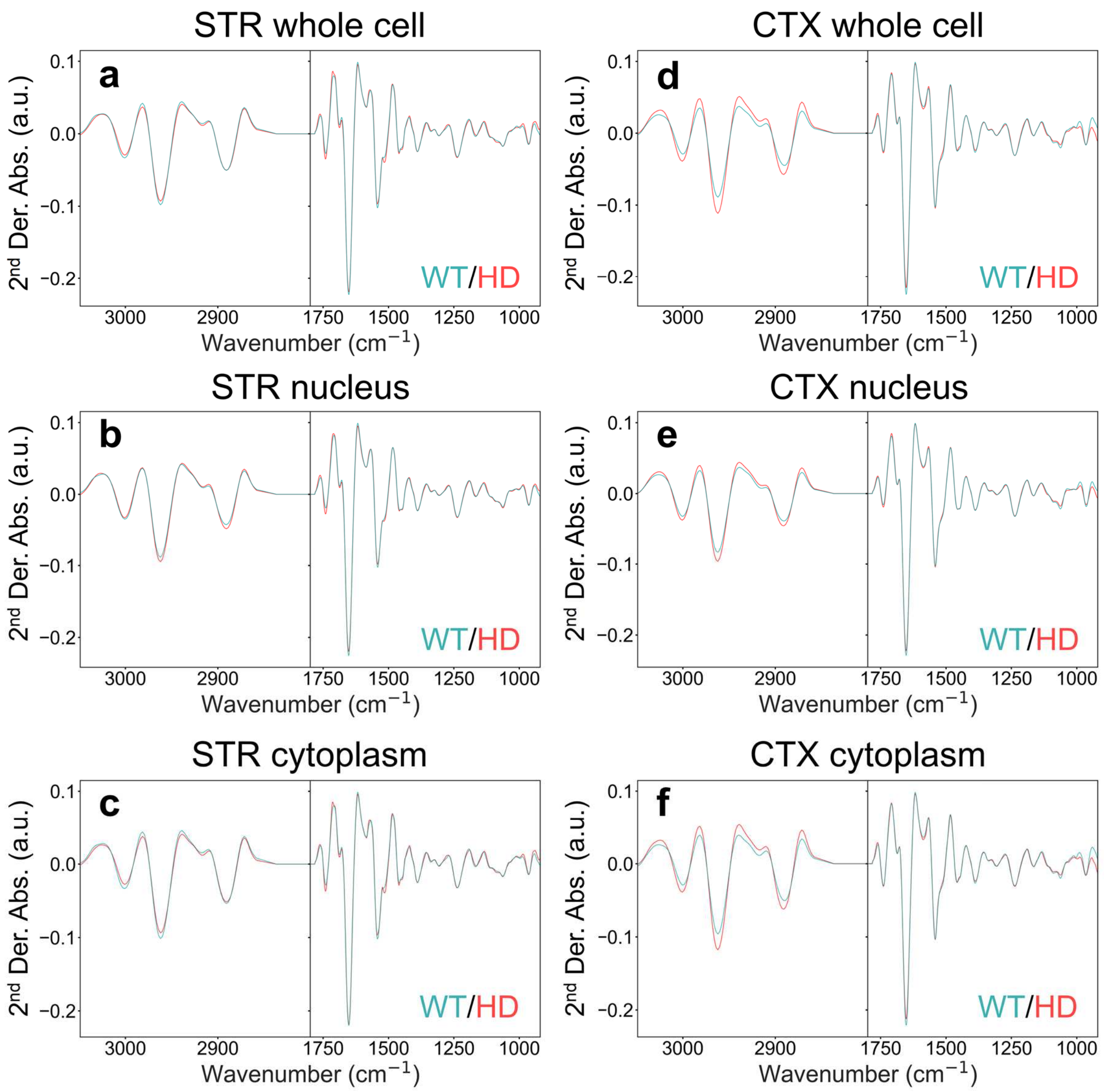

Supplementary Figure 2. Segmented cell spectra of striatum and cerebellum astrocytes. Whole cell, nucleus, and cytoplasm average spectra of WT and HD SV40T STR **(a-c)** and CTX **(d-f)** astrocytes. For visual purpose 2nd derivative normalized spectra are displayed between 3050-2800 cm^{-1} (lipid-rich region) and 1800-900 cm^{-1} ("fingerprint" region).

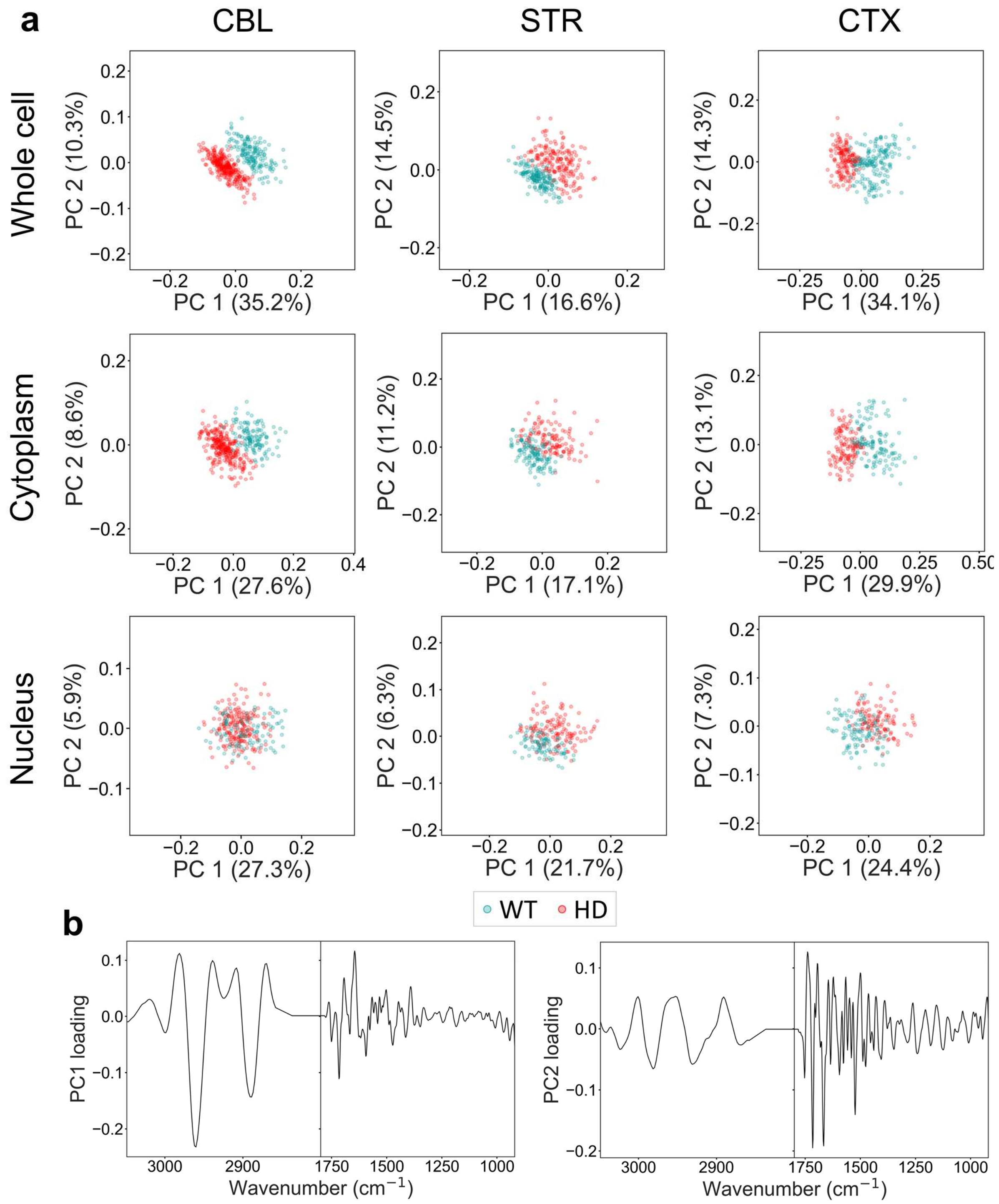

Supplementary Figure 3. PCA clustering distinguishes HD from WT for the three brain regions as in Fig. 5 (a) PCA plots corresponding to the UMAP analysis for the three brain regions performed in Fig. 3. **(b)** PC1 (left) and PC2 (right) loading for the WT and HD samples from the CBL whole cell PCA (top left corner). PC loadings show that lipid features (PC1 loading) and amide bands (PC2 loading) have a high contribution to the WT and HD cell discrimination.

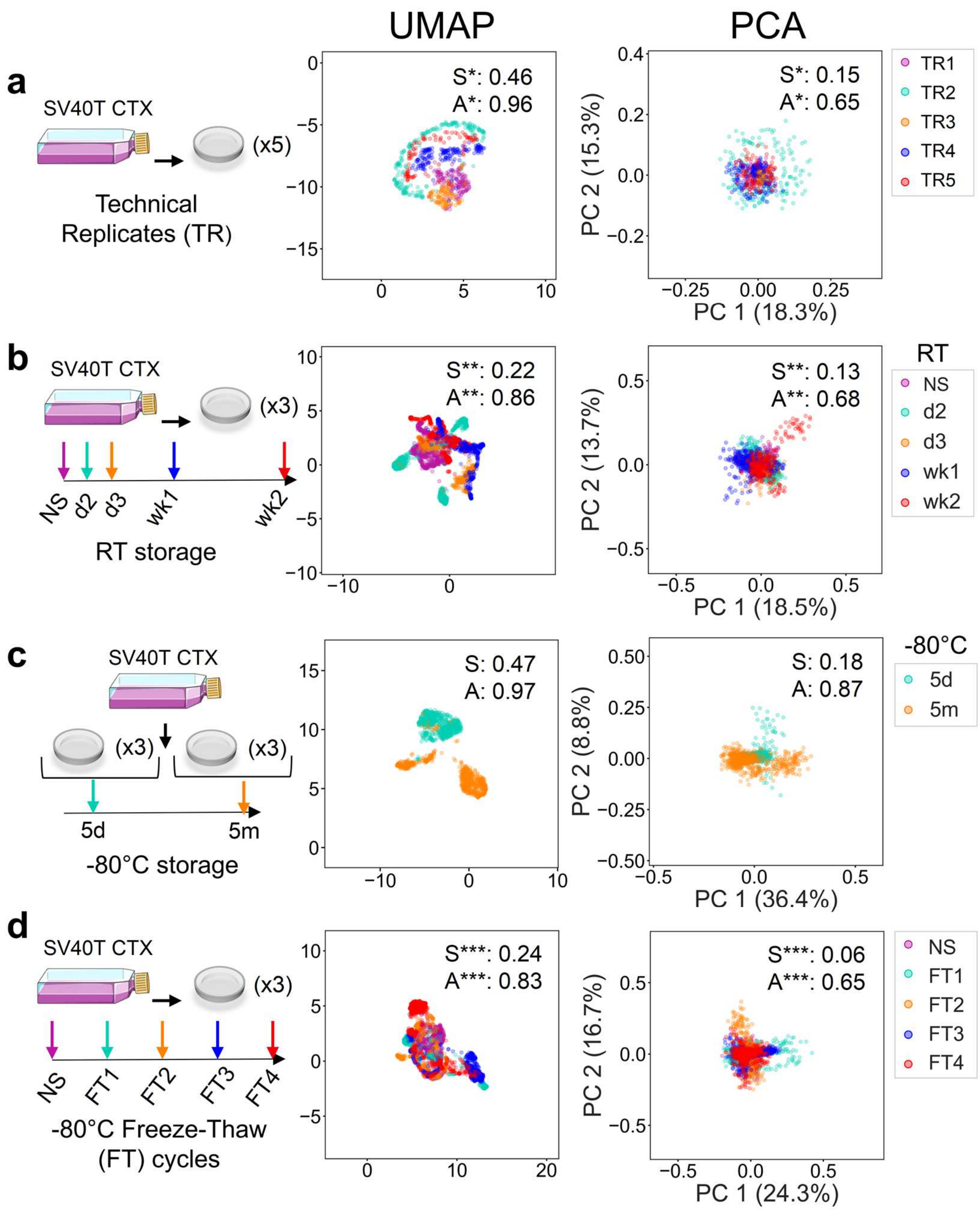

Supplementary Figure 4. Best practice conditions for reproducibility of the FTIR signatures measured under various conditions. Reproducibility of cell spectra under various conditions was assessed by UMAP (left) and PCA (right) analysis. **(a)** Technical replicates (TR) reproducibility. The S* and A* values are calculated for TR1 and TR5; **(b)** Storage at RT. The S** and A** values are calculated for NS (no storage) and wk2; **(c)** Storage at -80°C; the S and A values are calculated for 5 days (d) and 5 months (m); **(d)** Samples not stored (NS) compared to measurements after Freeze (-80°C) and thaw (RT) cycles. The S*** and A*** values calculated for NS and FT4.

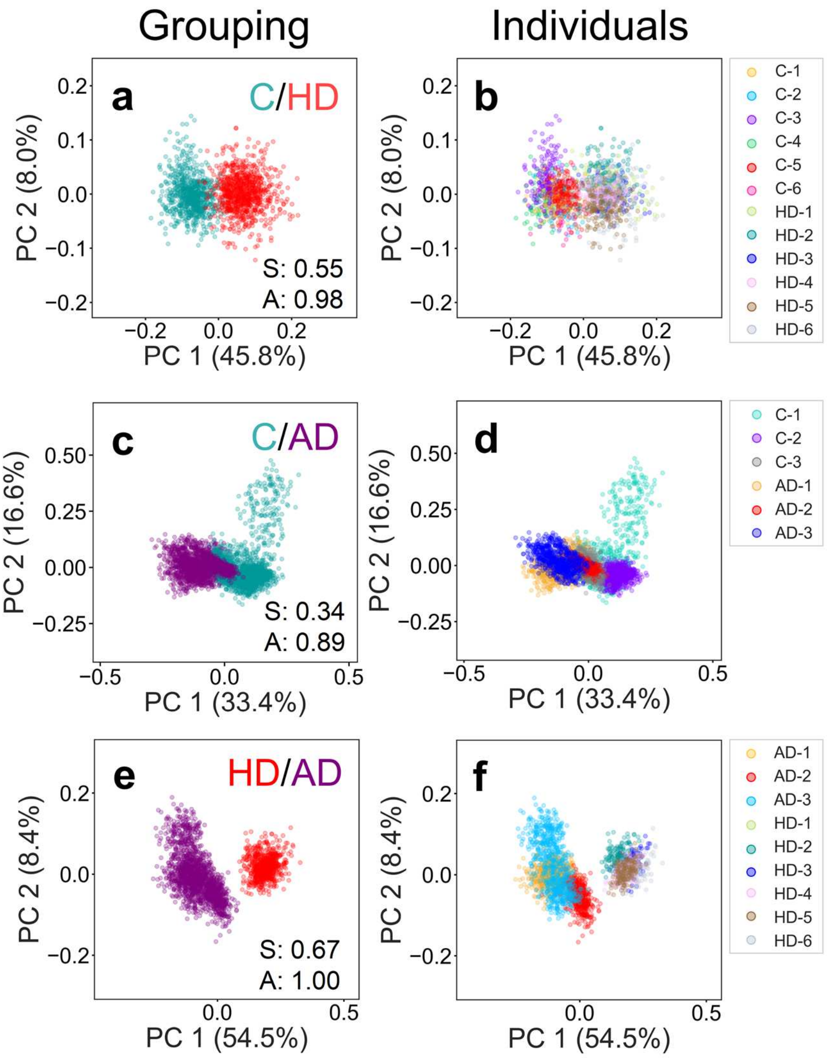

Supplementary Figure 5. The PCA analysis corresponding to the UMAP analysis (Fig. 8) for control and various disease fibroblast samples. FTIR spectra from human skin fibroblasts of controls (C) and Huntington's disease (HD, **a, b**) or Alzheimer's disease (AD, **c, d**) and HD *versus* AD (**e, f**) patients were evaluated by PCA. The PCA plots are the results of either pooled control or pooled disease samples (**a, c, e**), or displayed per individuals (**b, d, f**). All PCA analyses were performed on 2nd derivative normalized FTIR spectra of whole cells. S: silhouette score (*p*-value: <0.001), A: accuracy.

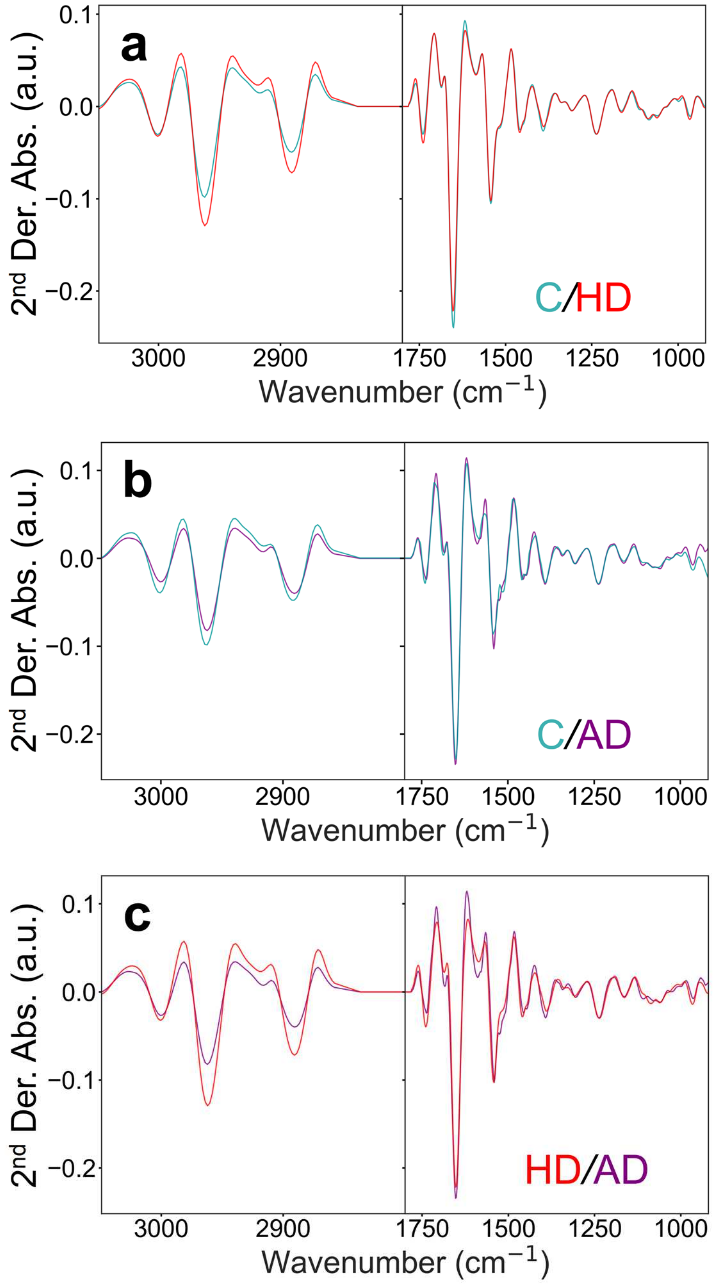

Supplementary Figure 6. HD and AD spectral signatures. Mean second derivative normalized FTIR spectra (whole cells) of HD **(a)** and AD **(b)** from Fig. 8 and supplementary Fig. 5, compared to the signature of control (C) cells. **(c)** Direct comparison of the HD and AD spectral signatures. For visual purpose 2nd derivative normalized spectra are displayed between 3050-2800 cm^{-1} (lipid-rich region) and 1800-900 cm^{-1} ("fingerprint" region).

Supplementary Table 1. Metrics for spectral classification (from Fig. 5; Supplementary Fig. 3).

	WT *vs* HD CBL			WT *vs* HD STR			WT *vs* HD CTX		
	NUC	CYT	CELL	NUC	CYT	CELL	NUC	CYT	CELL
S[a]	0.11*	0.45*	0.48*	0.13*	0.26*	0.33*	0.19*	0.37*	0.41*
Sens[b]	0.91	0.99	0.99	0.80	0.86	0.93	0.86	0.96	0.98
Spec[c]	0.27	0.93	0.98	0.67	0.88	0.97	0.70	0.88	0.92
A[d]	0.70	0.97	0.99	0.75	0.88	0.95	0.78	0.92	0.95

* *p*-value: <0.001. [a] S, silhouette score; [b] Sens, sensitivity; [c] Spec, specificity; [d] A, accuracy.

Supplementary Table 2. Demographics for disease patients and controls.

Label	Sample ID	Disease	Sex	Age[a] (yr)	Ethnicity	Cell origin	Brief description
HD-1	GM05030	HD	Male	56	Caucasian	NS	Choreic movements, dementia.
HD-2	GM05031	HD	Male	60	Caucasian	NS	Choreic movements, dementia.
HD-3	GM04476	HD	Male	57	Caucasian	NS	Onset at age 45, difficulty in ambulation with frequent falls.
HD-4	GM04691	HD	Male	31	Caucasian	NS	Onset at age 22, prominent laughing, involuntary vocalizations, dementia, dystonia.
HD-5	GM04777	HD	Male	53	Caucasian	NS	Clinically affected.
HD-6*	GM04693	HD	Male	33	Caucasian	NS	Onset at age 41.
AD-1	AG07377	AD	Male	60	Caucasian	Skin (Arm)	Progressive mental deterioration since age 51, development of aggressive behaviour. Moderate to marked cortical atrophy (CT scan). NFH.
AD-2	AG06262	AD	Male	66	Caucasian	Skin (Arm)	Progressive dementia with memory deficits, requiring hospitalization at age 62. Nonfocal cortical atrophy (CT scan, age 59). NFH.
AD-3	AG07376	AD	Male	60	Caucasian	Skin (Arm)	Progressive intellectual deterioration since age 54. Severe cerebral atrophy (CT scan). NFH.
C-1	AG08125	Control	Male	64	Caucasian	Skin (Arm)	Unaffected.
C-2	AG07623	Control	Male	60	Caucasian	Skin (Arm)	Unaffected.
C-3	AG08543	Control	Male	62	Caucasian	Skin (Arm)	Unaffected.
C-4	GM00288	Control	Male	64	Caucasian	Skin (Arm)	Unaffected.
C-5	GM09918	Control	Male	78	Caucasian	Skin (Arm)	Unaffected.
C-6	GM03658	Control	Male	68	Caucasian	Skin (Arm)	Unaffected.

All cell lines were obtained from the Coriell repository. [a] At sampling; NS: Not stated; NFH: no family history. * collected before the onset of symptoms.
